# Exploring targets of TET2-mediated methylation reprogramming as potential discriminators of prostate cancer progression

**DOI:** 10.1186/s13148-019-0651-z

**Published:** 2019-03-27

**Authors:** Shivani Kamdar, Ruth Isserlin, Theodorus Van der Kwast, Alexandre R. Zlotta, Gary D. Bader, Neil E. Fleshner, Bharati Bapat

**Affiliations:** 1 Lunenfeld-Tanenbaum Research Institute, Mount Sinai Hospital, 60 Murray Street, L6-304B, Toronto, ON M5T 3L9 Canada; 2 Department of Laboratory Medicine and Pathobiology, University of Toronto, Medical Sciences Building (6th floor), 1 King’s College Circle, Toronto, ON M5S 1A8 Canada; 3 Terrence Donnelly Centre for Cellular and Biomolecular Research, University of Toronto, 160 College St, Toronto, ON M5S 3E1 Canada; 4 Department of Pathology, University Health Network, University of Toronto, 200 Elizabeth St, Toronto, ON M5G 2C4 Canada; 5 Department of Surgery and Surgical Oncology, Division of Urology, University Health Network, University of Toronto, 190 Elizabeth St, Toronto, ON M5G 2C4 Canada

**Keywords:** Prostate cancer, TET2 knockout, Differential methylation profiling, Integrative analysis, Tumor progression

## Abstract

**Background:**

Global DNA methylation alterations are hallmarks of cancer. The tumor-suppressive TET enzymes, which are involved in DNA demethylation, are decreased in prostate cancer (PCa); in particular, TET2 is specifically targeted by androgen-dependent mechanisms of repression in PCa and may play a central role in carcinogenesis. Thus, the identification of key genes targeted by TET2 dysregulation may provide further insight into cancer biology.

**Results:**

Using a CRISPR/Cas9-derived *TET2*-knockout prostate cell line, and through whole-transcriptome and whole-methylome sequencing, we identified seven candidate genes—*ASB2*, *ETNK2*, *MEIS2*, *NRG1*, *NTN1*, *NUDT10*, and *SRPX*—exhibiting reduced expression and increased promoter methylation, a pattern characteristic of tumor suppressors. Decreased expression of these genes significantly discriminates between recurrent and non-recurrent prostate tumors from the Cancer Genome Atlas (TCGA) cohort (*n* = 423), and *ASB2*, *NUDT10*, and *SRPX* were significantly correlated with lower recurrence-free survival in patients by Kaplan-Meier analysis. *ASB2*, *MEIS2*, and *SRPX* also showed significantly lower expression in high-risk Gleason score 8 tumors as compared to low or intermediate risk tumors, suggesting that these genes may be particularly useful as indicators of PCa progression. Furthermore, methylation array probes in the TCGA dataset, which were proximal to the highly conserved, differentially methylated sites identified in our *TET2*-knockout cells, were able to significantly distinguish between matched prostate tumor and normal prostate tissues (*n* = 50 pairs). Except *ASB2*, all genes exhibited significantly increased methylation at these probes, and methylation status of at least one probe for each of these genes showed association with measures of PCa progression such as recurrence, stage, or Gleason score. Since *ASB2* did not have any probes within the *TET2*-knockout differentially methylated region, we validated *ASB2* methylation in an independent series of matched tumor-normal samples (*n* = 19) by methylation-specific qPCR, which revealed concordant and significant increases in promoter methylation within the *TET2*-knockout site.

**Conclusions:**

Our study identifies seven genes governed by *TET2* loss in PCa which exhibit an association between their methylation and expression status and measures of PCa progression. As differential methylation profiles and TET2 expression are associated with advanced PCa, further investigation of these specialized TET2 targets may provide important insights into patterns of carcinogenic gene dysregulation.

**Electronic supplementary material:**

The online version of this article (10.1186/s13148-019-0651-z) contains supplementary material, which is available to authorized users.

## Introduction

Prostate cancer (PCa) is the second most common cancer diagnosed in men worldwide, with significant global increases in incidence rates (2.4–21.4%) in 23 countries over the last 10 years [1, 2]. Widespread epigenomic dysregulation events in PCa have been identified as a hallmark of tumorigenesis [3–6]. Among these, tumor-specific DNA methylation (5mC) alterations and repression of gene expression are an emerging class of biomarkers as well as potential therapeutic targets, highlighting their importance in prostate carcinogenesis [7–14].

In PCa specifically, differential genomic patterns of 5mC and its derivative, 5-hydroxymethylcytosine (5hmC) have been linked to distinct molecular subtypes, indicating clear epigenetic stratification within tumors. For example, *TMPRSS2-ERG* fusion-positive tumors and tumors showing either *SPOP* or *FOXA1* mutations possess distinct methylation signatures as identified by unsupervised clustering [5], while significant loss of 5hmC is observed only within *ERG*-fusion negative PCa [15]. Our previous studies have shown that locus-specific 5hmC alteration is significantly correlated to transcriptional repression of multiple genes in prostate cancer cell lines, indicating the potential functional importance of these changes [16].In normal cells, the ten-eleven translocase (*TET*) family of genes oxidize 5mC to 5hmC, regulating levels of both epigenetic marks and promoting demethylation. The *TET* family consists of three members—*TET1*, *TET2*, and *TET3*—which generate 5hmC through Fe^2+^ and α-ketoglutarate-dependent dioxygenase activity [17, 18]. While all *TET* genes show loss of expression in PCa tissues, their mutation frequencies in primary prostate tumors are low, in contrast to the high mutation rates observed in metastatic castration-resistant prostate cancer and hematological malignancies [19–24].

Of the three *TET* enzymes, *TET2* in particular is uniquely implicated as having a central role in PCa biology due to its key involvement with androgen receptor (AR) signaling. *TET2* is able to bind to AR and its transcriptional coactivators in a prostate-specific interaction and has been linked to regulation of androgen-dependent genes such as PSA [23]. In turn, expression of the AR-induced microRNAs 29a and 29b specifically target and downregulate *TET2* in PCa, resulting in activation of AR and mTOR signaling pathways and promoting pro-carcinogenic biological functions [19, 20]. In contrast, its family member *TET3* has not been well investigated in the context of PCa and, although *TET1* is known to be co-recruited along AR as part of a hormonal response in normal prostate cells, this link has not yet been investigated in PCa [25]. Furthermore, loss of *TET2* activity has profound implications on PCa development, where reduced *TET2* expression is correlated with decreased disease-free survival, increased Gleason score, and metastasis [20, 23]. Thus, there is ample evidence to suggest that *TET2* loss may act as a key mechanism of PCa development, and exploration of its downstream target genes may provide new insights into cancer biology.

To investigate *TET2*’s role in PCa pathogenesis, we generated *TET2* knockout (KO) cell-lines in representative normal prostate cells to discover key candidate genes regulated by *TET2*-mediated methylation reprogramming. Here, we describe seven promising targets of epigenetic modification directed by *TET2* loss by analyzing their methylation and expression profiles in both prostate-derived cell lines and prostate tumors from the Cancer Genome Atlas (TCGA). These target genes are able to significantly differentiate between recurrent and non-recurrent tumors based on their expression status, further implicating *TET2-*governed changes as a significant process in carcinogenesis. Promoter methylation gain of one such promising candidate gene, ankyrin repeat and SOCS-box containing protein 2 (*ASB2*), within the *TET2*-target site is investigated in an independent series of primary prostate tumors. These studies elucidate the dynamics of *TET2*-mediated gene regulation in order to develop a novel selection strategy for identifying genes whose methylation and differential expression may play a key role in prostate carcinogenesis.

## Results

### Generation of *TET2*-knockout prostate cell lines

**Fig. 1:**
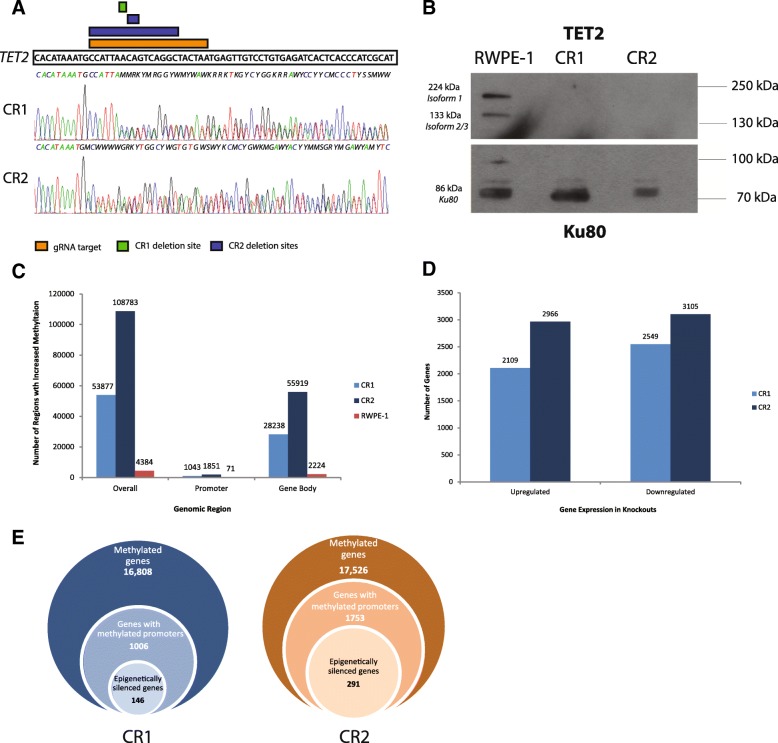
Methylation and expression profiles of CRISPR-Cas9 *TET2*-knockout cells. **a** Sanger sequencing chromatograms depict deletion sites observed in CR1 (green bar; heterozygous knockout) or CR2 (blue bar; functional homozygous knockout) which occur within the CRISPR guide RNA target site (orange bar). Mutant sequences for each knockout are shown, compared to the parental *TET2* sequence above. **b** Western blot shows complete loss of both *TET2* isoforms in CR1 and CR2 knockouts as compared to parental RWPE-1 cells (*top*). *Ku80* loading control is shown on the bottom. **c** Methylation levels are globally increased in *TET2*-knockout cells, with more differentially methylated regions (DMRs) in the promoter, gene body, and overall in both knockouts as compared to RWPE-1 (DiffBind, *p* < 0.05, *n* = 2). CR2 exhibits higher methylation levels as compared to CR1.The graph depicts the number of DMRs exhibiting increased methylation as compared to RWPE-1 (for CR1 and CR2) or as compared to either knockout (for RWPE-1). **d** Gene expression profiles show comparable levels of upregulation and downregulation in both knockouts, with 17.3% and 4.5% more genes showing significant downregulation than upregulation (1.5-fold change, *p* < 0.05). **e** Visual depiction of gene selection to identify genes exhibiting both significant methylation in the promoter region and significant loss of expression in either knockout (left, CR1; right, CR2) as compared to the total number of methylated genes

Sanger sequencing of clonally expanded populations using primers surrounding the indel target region (Additional file 1: Figure S1; Additional file 2: Table S1) revealed two CRISPR-knockout clones (CR1 and CR2; derived from the “P38” Sigma gRNA sequence [see the “Materials and methods” section for details]) exhibiting truncation mutations resulting in loss of both binding and catalytic domains [26]. CR1 showed heterozygous deletion of a single A nucleotide on one allele, resulting in a premature stop codon at 291aa, while CR2 showed a 17-bp deletion on one strand and a 2-bp deletion on another, resulting in functional homozygous knockout and stop codons present at 252aa or 256aa, respectively (Fig. 1a). Additional primer sequences were assessed to confirm that no off-target effects were observed (Additional file 2: Table S1, Additional file 3: Figure S2) as per the protocol described by Mali et al. [27]. Briefly, the last 13 bases of the gRNA sequence, along with the TGG protospacer sequence, were run through NCBI BLAST, and sequences with the highest similarity and including the protospacer sequence were assessed. The two sites tested for off-target effects possessed 93.8% sequence similarity (15/16 bases matching) to the BLASTed sequence. Complete loss of *TET2* protein expression in knockouts versus parental cells was confirmed via Western blot (Fig. 1b, Additional file 4: Figure S3).

### Methylation and expression profiles show extensive epigenetic silencing in *TET2*-knockout cells

We performed next-generation sequencing (NGS) of genomic DNA following methyl-binding protein capture (MBD-Seq) and poly-A enriched mRNA (RNA-seq) to determine the effects of *TET2* loss on prostate cells at both whole-methylome and whole-transcriptome levels. We observed large increases in differentially methylated regions (DMRs) in KOs versus parental cells (Fig. 1c, Additional file 5: Figure S4). Similarly, extensive changes in gene expression were observed in both KOs, with 17.3% and 4.5% more genes showing significant downregulation (> 1.5-fold decrease, *p* < 0.05) than upregulation (> 1.5-fold increase, *p* < 0.05) in CR1 and CR2, respectively (Fig. 1d, Additional file 6: Figure S5). 47.1% of downregulated genes and 42.3% of upregulated genes were shared between both *TET2* KO cell lines.

As promoter methylation gain is likely correlated with decreases in gene expression in cancer, especially in tumor suppressors [15–17], we examined the proportion of genes that showed both significant downregulation of expression (*p* < 0.05, edgeR v 3.8.6) and concomitant increased promoter methylation (*p* < 0.05, DiffBind), in either knockout (Fig. 1e), of which 36.7% were commonly shared between both KOs. We found that 14.5% (146 genes) of all genes exhibiting promoter methylation in CR1 also showed significant expression loss, which was comparable to the 16.6% of such genes (291 genes) discovered in CR2. A further 54 of these genes overlapped between both knockouts (Additional file 7: Table S2).

Subsequently, all genes exhibiting expression changes in either knockout were further assessed to determine their suitability as key targets of *TET2*-mediated reprogramming.

### A selected panel of key candidate genes exhibits differential methylation and downregulation in an independent cohort of primary prostate tumors

**Fig. 2:**
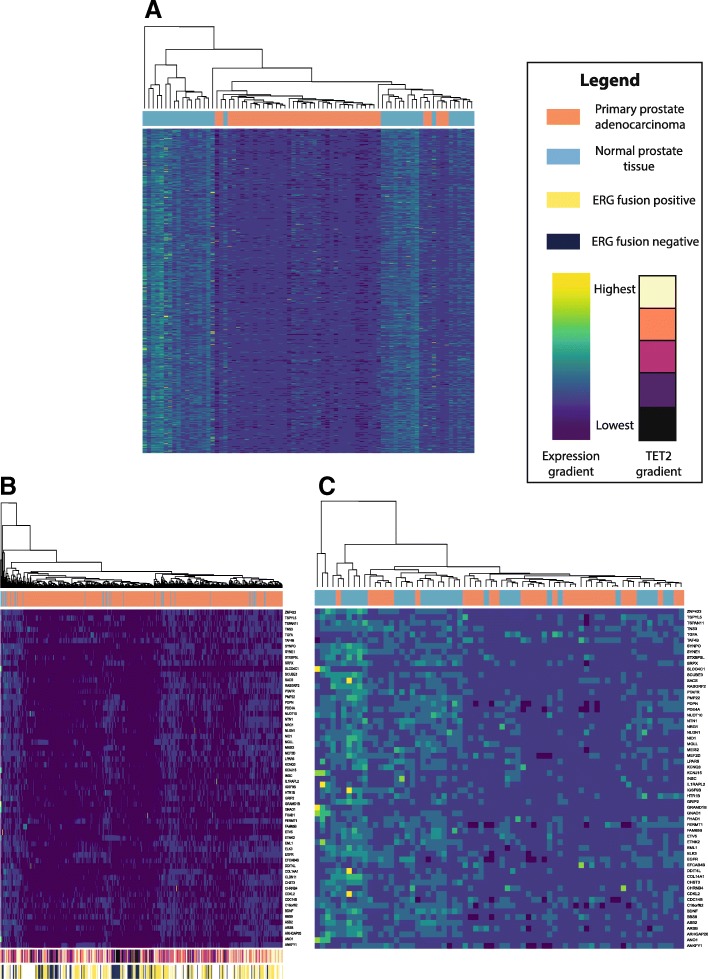
Genes exhibiting expression loss in *TET2-*knockout cell lines show discriminatory ability between normal prostate and prostate tumor based on expression status. Unsupervised heatmaps depict expression values normalized by gene for **a** 780 genes exhibiting significant loss of expression in both *TET2*-knockout cells (edgeR, *p* < 0.05) and a subset of tumors from the Cancer Genome Atlas (TCGA) within the lowest 10th percentile of *TET2* expression (Mann-Whitney *U*, *p* < 1.193E−5), on this low-*TET2* tumor subset; **b** 60 genes matching the above criteria and exhibiting increased promoter methylation in *TET2* knockouts (DiffBind, *p* < 0.05), in all TCGA tumors with expression data available (*n* = 423) or **c** in matched tumor and normal pairs (*n* = 35). Expression gradient bar indicates normalized expression levels, ranging from highest (yellow) to lowest (dark blue). *TET2* gradient bar indicates *TET2* expression in the entire TCGA dataset, ranging from highest (cream) to lowest (black). ERG fusion status is annotated in the entire TCGA dataset where data is available. Dendrograms indicate clustering between tissue samples

To identify the most significantly affected *TET2*-target genes with evidence of the highest potential impact in our model, we first compared global expression profiles in our cell line models to those reported in a subset of tumors from the TCGA which were in the bottom 10th percentile of *TET2* expression (*n* = 43 of 423 total tumors) as compared to normal prostate tissue from the same cohort (*n* = 35). Out of 4192 genes with significantly decreased expression in either *TET2* knockout (*p* < 0.05, FC < 0.75, edgeR; independent of methylation status), 780 genes exhibited significant loss of expression below the Bonferroni-corrected *p* value threshold of 1.193E−5 (Mann-Whitney *U* test) in the low-*TET2* TCGA subset (Fig. 2a, Additional file 8: Figure S6). Of these genes, 61 exhibited significant promoter methylation (*p* < 0.05, DiffBind) in our knockout cell lines, all of which had also been identified as exhibiting significant 5hmC loss in cancer cells from our previous study [16], implicating them as strong potential targets of *TET*-mediated demethylation (Additional file 9: Table S3). In turn, all but one of these genes exhibited significantly altered methylation in the low-*TET2* TCGA tumors with methylation array data available (*n* = 43) as compared to normal prostate samples (*n* = 50). Fourteen of these 60 genes exhibited significant association with ERG fusion status (Mann-Whitney *U* test, Bonferroni-corrected *p*-value < 0.05), of which 11 showed significantly increased expression in ERG-positive tumors and three showed significantly decreased expression (Additional file 10: Table S4). *TET2* expression was also significantly decreased in ERG-negative as compared to ERG-positive tumors (*p* = 7.124E−05, Mann-Whitney *U* test).

Next, we assessed the ability of these 60 *TET2*-target genes to distinguish between prostate tumor and normal samples (*n* = 35) expressing variable levels of *TET2*, in either all tumors with expression data available (*n* = 423, Fig. 2b) or in matched tumor and normal pairs only (*n* = 35, Fig. 2c). Twenty-seven genes exhibited significant loss of expression below the Bonferroni-corrected *p* value threshold (*p* < 4.2373E−4, Mann-Whitney *U* test) in both sets and were further assessed via pathway analysis.

### *TET2*-target genes are involved in critical biological processes governing signaling interactions and the immune system

We assessed significantly enriched biological pathway annotations (binomial *p* value < 0.05) for (a) all methylated and downregulated genes from our *TET2*-KO cells alone and (b) the 27 candidate genes identified above, with the Genomic Regions Enrichment of Annotations Tool (GREAT), using the whole genome as a background. Enriched pathways would not only highlight key functions significantly affected by global *TET2*-mediated alteration of the genes (set a), but would also provide insight into the potential biological consequences of candidate gene silencing (set b).

**Fig. 3:**
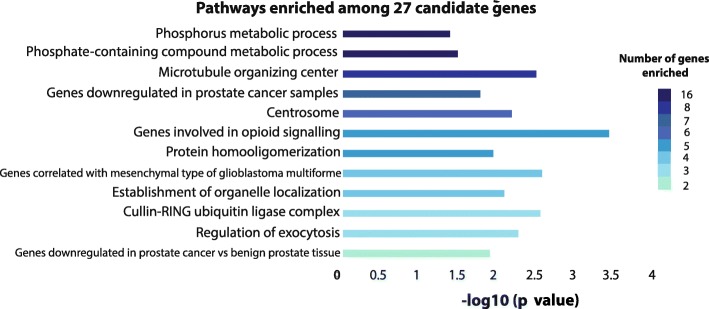
Pathway analysis of candidate genes significantly altered by *TET2*-knockout. Selected, significant pathway enrichment annotations from GREAT for genes exhibiting significantly altered expression in both *TET2*-knockout cell lines and in tumor versus normal samples from the TCGA (binomial *p* value < 0.05). Bar coloration indicates the number of candidate genes enriched within each pathway

Among all silenced genes in our knockouts (set a), many of the pathways enriched were associated with immune functionality, cellular adhesion, and cell death, especially with regards to the formation of inflammasomes, oligomeric complexes involved in inflammatory cytokine generation, and cell death (Additional file 11: Figure S7). When examining the panel of 27 candidate genes (set b), a differential pathway clustering profile enriched for pathways including phosphate metabolism, microtubule organization, cell-cell signaling, and DNA helicase activity was observed (Fig. 3). Interestingly, five of the 27 candidate genes identified above (*ANO1*, *MEIS2*, *PDE4A*, *PMP22*, and *SRPX*) were identified through pathway analysis as genes known to be downregulated in prostate cancer samples specifically, indicating the relevance of these candidates to PCa in other datasets as well.

### Expression levels and methylation status of key *TET2*-target genes exhibit correlation with measures of prostate cancer progression

As reduced *TET2* levels are correlated with advanced PCa and decreased survival [19, 22], we assessed the ability of changes in expression of our 27 *TET2*-target candidate genes to discriminate based on pathological stage, Gleason score (GS), and recurrence in the TCGA dataset (*n* = 423 tumor samples). Lowered expression of nine of these genes was able to significantly distinguish recurrent tumors from non-recurrent tumors (Mann-Whitney *U* test, *p* < 0.05, Table 1). Five genes could distinguish between tumors of differing pathological stage and Gleason scores. Of these, ankyrin repeat and SOCS box protein 2 (*ASB2*), Meis homeobox 2 (*MEIS2*), and sushi repeat-containing protein X-linked (*SRPX*) were able to specifically distinguish GS6 or GS7 from GS8 or higher tumors (Bonferroni-adjusted Dunn test, *p* < 0.05).

**Table 1:** Significant changes in TET2-target gene expression associated with prostate cancer development and progression

Gene	Tumor vs normal^1^ Matched tumor and normal	Tumor vs normal^1^ All TCGA tumors	Recurrence^1^	Stage^1^	Gleason score (overall)^2^	GS6 vs GS7^3^	GS6 vs GS8 +^3^	GS7 vs GS8 +^3^
ASB2	**2.9543E−08**	**1.1163E−10**	**0.0189**	**0.0095**	**3.1843E−05**	0.17	**0.00044413**	**2.1003E−4**
ETNK2	**8.0425E−15**	**1.6991E−16**	**0.0147**	0.3725	0.1526	1	0.4005	0.0924
KCNJ15	**1.1797E−09**	**6.9856E−14**	**0.0436**	0.6534	0.0842	0.4247	1	**0.0487**
MEIS2	**8.6435E−09**	**1.4128E−13**	**0.0125**	**0.0256**	**0.0010**	0.5208	**0.0100**	**0.0015**
NRG1	**1.6456E−05**	**1.9533E−11**	**0.0013**	**0.0023**	**0.0016**	1	0.0918	**6.6543E−4**
NTN1	**3.1531E−05**	**1.1782E−07**	**0.0091**	**0.0261**	**0.0273**	1	0.205	**0.0139**
NUDT10	**2.4896E−08**	**2.8735E−10**	**0.0235**	0.5739	0.4542	0.4667	0.3151	0.9259
PDE4A	**2.1250E−09**	**1.2586E−12**	**0.0431**	0.6292	0.5444	0.9831	1	0.4186
SRPX	**1.0079E−05**	**3.2133E−08**	**0.0078**	**0.0014**	**1.1851E−07**	1	**0.0014**	**8.9930E−08**

Bolded and underlined numbers represent significant *p* values as derived from ^1^Mann-Whitney *U* test, ^2^Kruskal-Wallis test, and ^3^Bonferroni-adjusted Dunn test

**Fig. 4:**
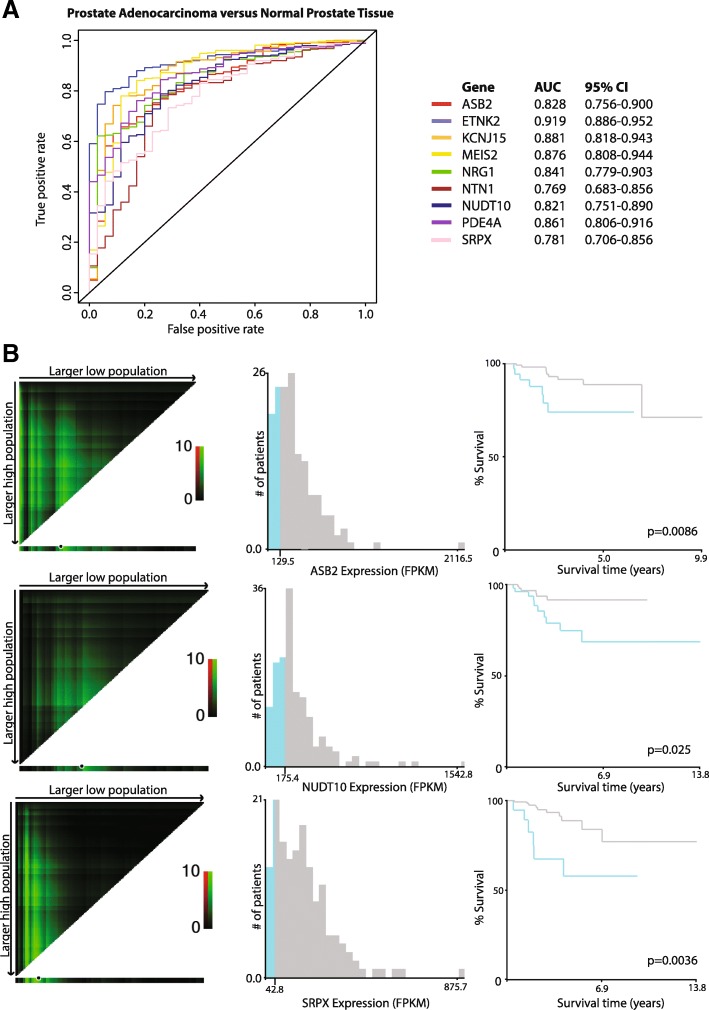
Candidate gene expression is indicative of tumor status and can predict worse recurrence-free survival in patients. **a** Receiver operating characteristic (ROC) curves for individual candidate gene expression, stratifying between benign (*n* = 35) and tumor (*n* = 423) patients in the TCGA cohort. AUCs and 95% confidence intervals for each gene are provided on the right. **b** X-tile analysis and Kaplan-Meier plots for prediction of biochemical recurrence-free survival in the TCGA cohort. Left: X-tile plots depict *χ*^2^ values for all possible data divisions, with brightness indicating strength of association and green coloration indicating a direct relationship. Black circles on the bottom bars for each graph depict automatically generated cut points maximizing the *χ*^2^ value in an auto-generated training set. Middle: Histogram depicting the number of patients in the auto-generated validation set below (blue) or above (gray) the cutoff point. Right: Kaplan-Meier plot showing recurrence-free survival in low-expressing (blue, below cutoff) or high-expressing (gray, above cutoff) groups for each gene. Log-rank *p* values are indicated on each graph

We next examined the performance of these nine genes’ expression levels in discriminating between prostate tumor and normal tissue through receiver operating curve (ROC) analysis in the TCGA cohort (Fig. 4a, Table 2). All genes exhibited strong performance, with *ETNK2* expression providing the highest accuracy of classification (AUC = 0.919, 95% CI 0.886–0.952; sensitivity = 0.797, specificity = 0.943). However, although all nine genes were significant predictors of tumor status in univariate logistic regression analyses, four genes in particular—*MEIS2*, *NRG1*, *NTN1*, and *NUDT10*—were determined to be independent predictors by multivariate logistic regression (Table 3).

**Table 2:** Sensitivity and specificity for nine candidate genes in classifying prostate tumor vs normal

Gene	Sensitivity	Specificity
ASB2	0.631	0.914
ETNK2	0.797	0.943
KCNJ15	0.778	0.886
MEIS2	0.839	0.829
NRG1	0.619	0.971
NTN1	0.749	0.771
NUDT10	0.787	0.743
PDE4A	0.759	0.829
SRPX	0.73	0.714

**Table 3:** Univariate and multivariate logistic regression analyses for classification of prostate tumor vs normal prostate

Gene	Univariate *p* value	Univariate odds ratio	Univariate confidence interval	Multivariate *p* value	Multivariate odds ratio	Multivariate confidence interval
ASB2	2.05E−08***	0.998	0.997–0.998	0.96	1	0.998–1.002
ETNK2	2.34E−13***	0.99	0.987–0.993	0.1	0.996	0.992–1.001
KCNJ15	1.50E−10***	0.983	0.978–0.988	0.17	0.995	0.988–1.002
MEIS2	7.25E−14***	0.997	0.997–0.998	0.019*	0.998	0.997–1.000
NRG1	2.07E−08***	0.97	0.959–0.980	0.028*	0.98	0.964–0.998
NTN1	2.08E−06***	0.997	0.995–0.998	0.028*	1.004	1.000–1.007
NUDT10	1.26E−05***	0.998	0.997–0.999	0.010*	0.999	0.998–1.000
PDE4A	3.37E−10***	0.993	0.991–0.995	0.064	0.997	0.993–1.000
SRPX	1.42E−07***	0.994	0.992–0.997	0.89	1	0.997–1.003

Statistical significance indicated by asterisks: *0.01 < *p* < 0.05; **0.001 < *p* < 0.01; ***p < 0.001

Note: odds ratios are modest due to the large scale range of the data (see Fig. 4b)

As Mann-Whitney *U* analysis indicated that the expression of the nine candidate genes was correlated with recurrence, we assessed whether decreased gene expression was correlated with recurrence-free survival in PCa through X-tile and Kaplan-Meier analysis. Lowered expression of three of the nine genes—*ASB2*, *NUDT10*, and *SRPX*—was significantly correlated with poor prognosis in PCa patients (Fig. 4b), while two more genes—*ETNK2* and *NTN1*—were trending (*p* < 0.10, Additional file 12: Figure S8). Overall, these analyses exhibited the utility of these genes as promising indicators of PCa status and progression.

**Fig. 5:**
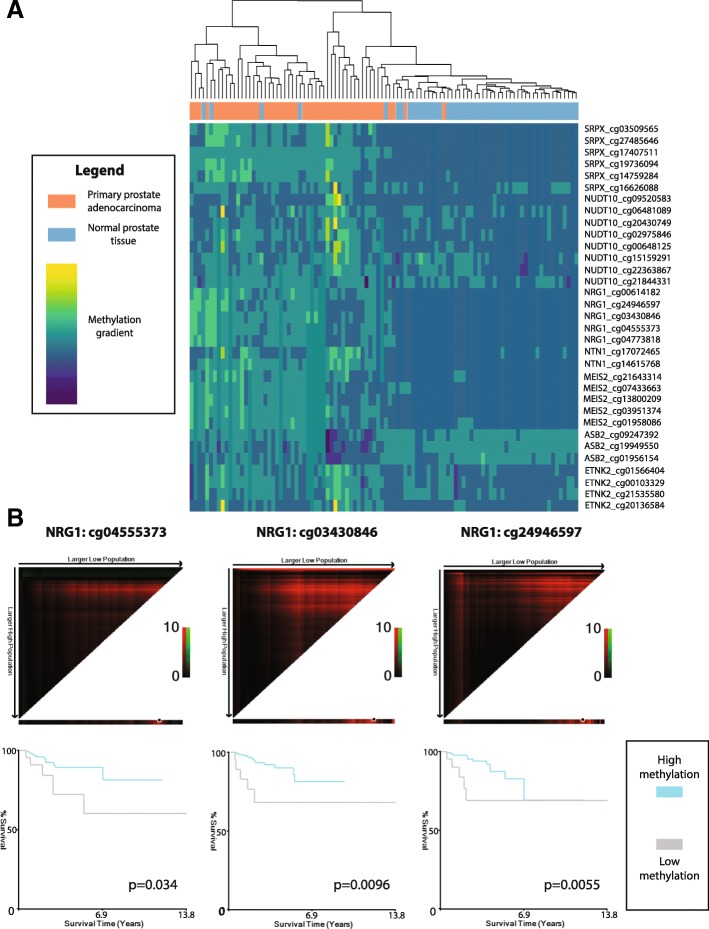
Tumor methylation comparison of candidate genes and predictive ability for recurrence. **a** Unsupervised heatmap depicting methylation beta values normalized by probe for seven genes exhibiting significantly altered expression and methylation in both knockouts and matched prostate tumor and normal samples (*n* = 50). Methylation gradient bar indicates normalized methylation levels, ranging from highest (yellow) to lowest (dark blue). Dendrograms indicate clustering between tissue samples. **b** X-tile analysis depicting methylation probes significantly associated with outcome. High-methylation probe status (gray, above cutoff) was indicative of worse recurrence-free survival as compared to patients with low-methylation probe status (blue, below cutoff) for the three probes shown. Log-rank *p* values are indicated on each graph

We subsequently examined methylation of the nine candidate genes able to distinguish recurrent tumors in the TCGA database at sites proximal to (± 500 bp) or within regions gaining methylation in our KO cell line models to assess possible linkages between the *TET2*-target gene panel and PCa. Out of the nine genes, seven possessed TCGA probes proximal to our *TET2*-KO differential methylation sites which showed discriminatory ability between matched tumor and normal samples (*n* = 50 pairs) based on differential methylation values at specific CpG sites (Table 4, Fig. 5a). Methylation of *SRPX* was found to be completely independent of ERG fusion status in tumors. *ASB2*, *MEIS2*, and *NRG1* methylation was increased in ERG-fusion positive tumors, while *ETNK2*, *NTN1*, and *NUDT10* showed decreased methylation associated with ERG status (Additional file 13: Table S5). Gene probe methylation differences were validated in an independent methylation array dataset (*n* = 90; accession number GSE73549), with probes in all genes except for *ASB2* able to significantly distinguish between normal tissues, prostate tumors, and lymph node PCa metastases (Kruskal-Wallis test *p* < 0.05, Additional file 14: Table S6). Increased methylation of two probes in *MEIS2* (cg01566404 and cg13800209) was also able to significantly distinguish between prostate tumor sites and metastatic cancer (Bonferroni-adjusted Dunn test, *p* < 0.05).

**Table 4:** Significant changes in TET2-target gene methylation associated with prostate tumor development and progression

CpG location	Tumor vs normal^1^	Recurrence^1^	Stage^1^	Gleason score (overall)^2^	GS6 vs GS8 +^3^	GS7a vs GS8 +^3^	GS7b vs GS8 +^3^	GS6 vs normal^3^	GS7a vs normal^3^	GS7b vs normal^3^	GS8+ vs normal^3^
	*ASB2 (chr14)*										
94423399	5.3192E−04***	0.2409	0.9747	1.2538E−04***	1	0.1919	1	0.0416*	0.0151*	5.4253E−04***	2.6764E−05***
94423704	5.4896E−07***	0.702	0.658	1.9181E−12***	1	0.258	1	1.0501E−04***	1.4758E−08***	1.8719E−10***	6.6097E−13***
94424156	0.0011**	0.7441	0.1885	1.6348E−06***	1	0.496	1	0.0481*	5.4625E−04***	1.5358E−06***	1.5159E−06***
	*ETNK2 (chr1)*										
204121503	0.0012**	0.0306*	0.4185	1.3158E−12***	0.0431*	2.2029E−05***	0.2733	0.0150*	3.8371E−04***	4.8206E−07***	3.5387E−13***
204121899	0.00017425***	0.0618	0.0142*	6.6353E−06***	0.7984	9.3204E−04***	1	0.1681	0.3024	0.0013**	9.6444E−06***
204121923	1.0324E−08***	0.2862	0.0518	3.5270E−10***	1	0.1555	1	3.4274E−04***	2.0944E−06***	1.0047E−08***	1.0578E−10***
204121925	3.4330E−07***	0.0649	0.0044**	7.8498E−11***	1	0.0048**	1	2.4590E−04***	8.0568E−05***	2.0361E−08***	3.9094E−11***
	*MEIS2 (chr15)*										
37389951	1.0768E−09***	0.133	0.0183*	2.7510E−16***	1	0.3942	0.4735	3.4941E−07***	1.2511E−12***	2.1070E−10***	2.9902E−17***
37390176	3.9592E−12***	0.1417	0.0437*	6.0747E−20***	0.7401	0.1042	0.3351	1.5444E−07***	1.7809E−14***	8.8326E−13***	3.6691E−21***
37390284	5.5646E−12***	0.6529	0.0347*	2.1732E−18***	1	0.4868	0.4465	2.7653E−08***	1.7689E−14***	1.2108E−11***	4.3682E−19***
37390326	1.7682E−10***	0.9925	0.0507	1.0213E−15***	1	1	1	1.7273E−06***	3.7762E−14***	1.0088E−11***	3.4463E−15***
37390666	9.9208E−12***	0.781	0.4833	1.2698E−14***	1	1	1	9.1587E−06***	1.2821E−13***	2.0310E−11***	1.1459E−13***
	*NRG1 (chr8)*										
31496966	8.6752E−10***	0.6985	0.0150*	2.5631E−14***	1	1	1	1.9238E−04***	1.2260E−14***	1.8753E−10***	4.0103E−12***
31497042	5.0501E−12***	0.0647	0.0319*	2.2705E−19***	1	0.2969	1	2.0176E−08***	2.0255E−14***	3.9698E−14***	9.9203E−20***
31497082	4.0886E−11***	0.0448*	0.0057**	2.1494E−17***	1	0.5099	1	6.7545E−07***	1.7471E–13***	8.9222E−13***	7.0663E−18***
31497464	1.4066E−12***	0.0578	0.0021**	6.4985E−19***	1	1	1	1.1607E−07***	8.3519E−17***	1.7644E−12***	1.4988E−18***
31498006	9.9208E−12***	0.2202	0.0025**	1.1949E−17***	0.1303	1	1	2.4495E−04***	6.1828E−14***	2.4410E−14***	8.2690E−17***
	*NTN1 (chr17)*										
8924093	1.6691E−13***	0.0165*	0.3672	2.4340E−21***	0.205	0.0761	1	1.9637E−06***	1.6375E−14***	2.2252E−16***	1.3727E−21***
8924309	1.0069E−07***	0.3684	0.3741	8.9654E−04***	1	1	0.6196	1	0.0044**	2.0804E−04***	0.0105*
	*NUDT10 (chrX)*										
51074836	4.7839E−06***	0.2173	0.1356	0.0016**	1	1	1	0.0108*	0.0031**	7.0332E−04***	0.0098**
51074936	1.8063E−06***	0.9068	0.985	9.0493E−07***	1	1	1	0.0023**	1.3634E−05***	8.6805E−06***	1.8934E−07***
51074953	0.0140*	0.8895	0.7916	0.2201	1	1	1	0.5458	0.1431	0.3771	0.1566
51075046	8.3171E−06***	0.9564	0.1815	1.1351E−05***	1	0.4569	1	0.0936	0.0082**	3.7804E−06***	4.5893E−05***
51075126	3.3740E−05***	0.2602	0.4731	7.1943E−06***	0.9654	1	0.0695	0.0017**	2.2205E−04***	1.3429E−06***	0.0035**
51075273	0.0020**	0.9452	0.6984	0.0230*	1	1	0.1072	1	1	*0.0217	1
51075797	0.0244*	0.8575	0.3457	0.275	1	1	1	0.4945	0.2262	0.3023	0.2772
51076119	0.0022**	0.0123*	0.0065**	6.5620E−09***	0.6303	3.5148E−05***	0.1189	0.0123*	0.0322*	5.7175E−04***	3.0404E−09***
	*SRPX (chrX)*										
38080219	0.0253*	0.5836	0.2111	3.8518E−09***	1	8.1850E−04***	0.076	0.0022**	0.0013**	2.4744E−04***	2.8109E−10***
38080303	6.5280E−14***	0.3153	0.0722	3.5526E−19***	0.1348	0.3735	1	4.2450E−05***	5.8355E−14***	2.3822E−15***	7.1231E−19***
38080327	4.5130E−14***	0.1105	0.0488*	1.6149E−21***	0.1261	0.05907	1	2.8937E−06***	9.2431E−15***	1.4417E−15***	3.4189E−22***
38080364	1.5537E−12***	0.0242*	0.1777	1.8382E−19***	0.2859	0.2103	1	4.3763E−06***	3.3823E−14***	1.6902E−14***	6.1925E−20***
38080412	2.6758E−12***	0.9925	0.0321*	3.0305E−19***	0.2321	0.0186*	1	5.9957E−06***	4.7647E−12***	2.7680E−14***	4.8523E−20***
38080425	5.4179E−13***	0.9117	0.0162*	5.9541E−19***	0.3035	0.1457	1	1.0253E−05***	6.8240E−13***	1.7779E−15***	7.6889E−19***

*p* values derived from ^1^Mann-Whitney *U* test, ^2^Kruskal-Wallis test, and ^3^Bonferroni-adjusted Dunn test

Statistical significance indicated by asterisks: *0.01 < *p* < 0.05; **0.001 < *p* < 0.01; ***p < 0.001

NA values for Dunn tests indicate lack of significance for Kruskal-Wallis test

**Fig. 6:**
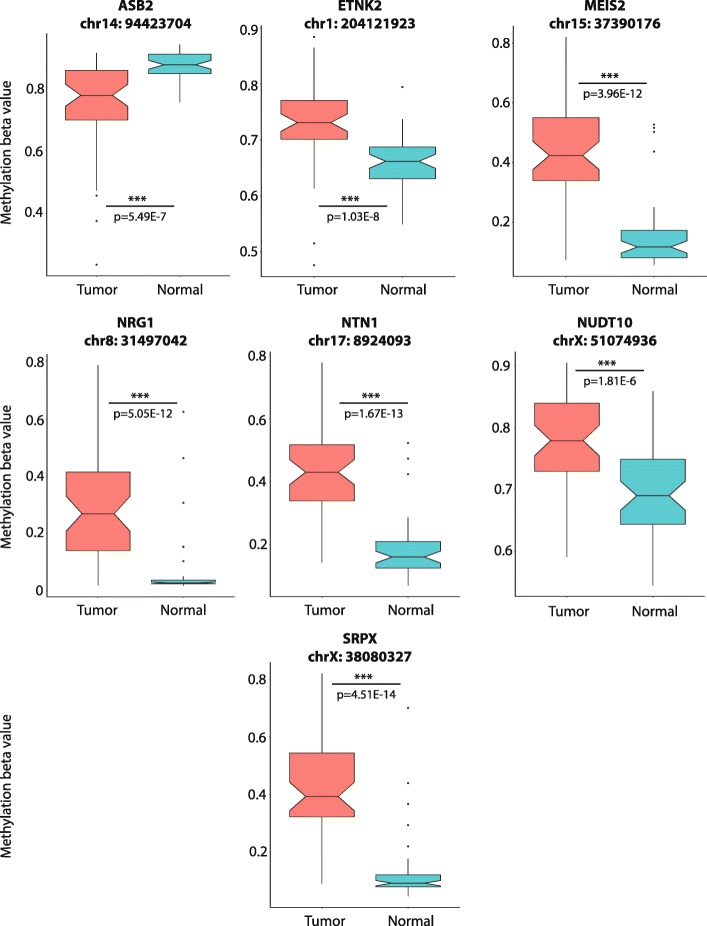
Methylation differences between tumor and normal samples for seven candidate genes. Notched boxplots show distribution of methylation beta values from 450 k methylation array for representative methylation probes within 500 bp surrounding the differentially methylated regions observed in candidate genes from our *TET2* knockouts in matched tumor vs normal samples (*n* = 50). Notches indicate 95% confidence interval for medians. Significance for aggregated values determined by Mann-Whitney *U* test

All genes except *ASB2* exhibited significantly increased tumor methylation as compared to normal prostate in all significant proximal probes, while *ASB2* methylation was significantly decreased in TCGA prostate tumors at all significant probes within the promoter as well as its single proximal probe (Additional file 15: Figure S9, Table 5, Fig. 6). These findings were reflected in univariate logistic regression analyses of logistic-transformed methylation *β*-values (*M*-values; Table 6) in the entire TCGA dataset (*n* = 478), where *ASB2* methylation probes were unique in exhibiting significant association with tumor status based on decreased, rather than increased, methylation values (ORs ranging from 0.09 to 0.30). Intriguingly, *ASB2* did not possess any probes located within the *ASB2* promoter methylation peak from the *TET2*-KO differentially methylated site (DMS), raising the possibility that the increased methylation observed in *TET2* KO cells was specific to that region. Most methylation probes exhibited strong discriminatory performance between tumor and normal tissue, with median AUC of 0.834 and probes within three genes—*MEIS2*, *NRG1*, and *SRPX—*exhibiting sensitivities greater than 90% (Table 5). However, multivariate logistic regression analyses indicated that probes in *ETNK2* (cg20136584, Wald *p* value = 0.0014) and *NRG1* (cg00614182, Wald *p* value = 0.049) were independent predictors of tumor versus normal status in the TCGA cohort, indicating that increased methylation of these genes may be particularly important in PCa (Table 6).

**Table 5:** Sensitivity and specificity of candidate gene methylation in classifying prostate tumor vs normal prostate tissue

Gene	Probe ID	Sensitivity	Specificity	AUC
ASB2	cg01956154	0.514	0.8	0.684
ASB2	cg19949550	0.759	0.8	0.834
ASB2	cg09247392	0.61	0.84	0.747
ETNK2	cg20136584	0.61	0.92	0.803
ETNK2	cg21535580	0.486	0.9	0.687
ETNK2	cg00103329	0.612	0.94	0.798
ETNK2	cg01566404	0.703	0.86	0.79
MEIS2	cg01958086	0.811	0.84	0.879
MEIS2	cg03951374	0.86	0.92	0.917
MEIS2	cg13800209	0.914	0.84	0.909
MEIS2	cg07433663	0.79	0.88	0.881
MEIS2	cg21643314	0.867	0.82	0.871
NRG1	cg04773818	0.794	0.9	0.849
NRG1	cg04555373	0.902	0.88	0.912
NRG1	cg03430846	0.888	0.86	0.891
NRG1	cg24946597	0.855	0.92	0.914
NRG1	cg00614182	0.862	0.84	0.881
NTN1	cg14615768	0.839	0.94	0.926
NTN1	cg17072465	0.481	0.94	0.671
NUDT10	cg21844331	0.381	0.86	0.62
NUDT10	cg22363867	0.755	0.7	0.747
NUDT10	cg15159291	0.341	0.86	0.61
NUDT10	cg00648125	0.558	0.84	0.712
NUDT10	cg02975846	0.558	0.88	0.705
NUDT10	cg20430749	0.311	1	0.558
NUDT10	cg06481089	0.306	0.92	0.603
NUDT10	cg09520583	0.5	0.92	0.75
SRPX	cg16626088	0.762	0.66	0.771
SRPX	cg14759284	0.818	0.92	0.902
SRPX	cg19736094	0.895	0.9	0.925
SRPX	cg17407511	0.874	0.9	0.909
SRPX	cg27485646	0.902	0.84	0.905
SRPX	cg03509565	0.8	0.92	0.905

**Table 6:** Univariate and multivariate logistic regression analyses for classification of prostate tumor vs normal prostate based on probe methylation

Gene	Probe	Univariate *p* value	Univariate odds ratio	95% confidence interval	Multivariate *p* value	Multivariate odds ratio	95% confidence interval
ASB2	cg01956154	3.48E−05***	0.3	0.17–0.53	0.72	1.46	0.18–11.84
ASB2	cg19949550	5.37E−11***	0.18	0.11–0.30	0.09	0.3	0.070–1.20
ASB2	cg09247392	4.42E−07***	0.09	0.04–0.24	0.18	0.12	0.010–2.60
ETNK2	cg20136584	4.89E−09***	16.64	6.49–42.68	**0.0014	154.88	6.95–3449.78
ETNK2	cg21535580	6.27E−05***	3.17	1.80–5.59	0.08	19.76	0.70–559.54
ETNK2	cg00103329	1.09E−08***	8.27	4.01–17.07	0.26	11.95	0.15–933.7
ETNK2	cg01566404	1.38E−08***	5.69	3.12–10.36	0.17	0.09	0.00–2.78
MEIS2	cg01958086	1.12E−14***	2.85	2.19–3.72	0.73	0.81	0.24–2.70
MEIS2	cg03951374	< 2.00E−16***	3.88	2.84–5.29	0.77	0.81	0.20–3.33
MEIS2	cg13800209	2.08E−12***	2.57	1.97–3.34	0.064	1.88	0.96–3.66
MEIS2	cg07433663	< 2.00E−16***	2.35	1.92–2.88	0.46	0.75	0.35–1.60
MEIS2	cg21643314	8.79E−13***	3.77	2.62–5.43	0.71	0.79	0.23–2.72
NRG1	cg04773818	3.41E−14***	2.08	1.72–2.52	0.67	0.85	0.40–1.80
NRG1	cg04555373	3.96E−14***	3.18	2.35–4.28	0.071	4.67	0.88–24.94
NRG1	cg03430846	2.84E−14***	3.5	2.54–4.84	0.31	0.44	0.090–2.11
NRG1	cg24946597	2.41E−11***	2.82	2.08–3.83	0.81	0.9	0.37–2.16
NRG1	cg00614182	1.16E−13***	3.76	2.65–5.34	*0.049	2.7	1.70–26.00
NTN1	cg14615768	< 2.00E−16***	6.12	4.03–9.27	0.46	0.53	0.10–2.83
NTN1	cg17072465	0.000213***	3.25	1.74–6.08	0.36	0.32	0.030–3.71
NUDT10	cg21844331	0.14	1.37	0.90–2.09	NA	NA	NA
NUDT10	cg22363867	4.44E−07***	3.28	2.07–5.21	0.3	0.37	0.060–2.41
NUDT10	cg15159291	0.02*	2.82	1.18–6.78	0.35	0.18	0.00–6.51
NUDT10	cg00648125	4.38E−05***	2.94	1.75–4.93	0.9	0.91	0.21–3.98
NUDT10	cg02975846	2.53E−05***	2.7	1.70–4.29	0.19	3.84	0.52–28.62
NUDT10	cg20430749	0.023*	1.51	1.06–2.15	0.23	0.32	0.050–2.05
NUDT10	cg06481089	0.13	0.65	0.37–1.13	NA	NA	NA
NUDT10	cg09520583	6.25E−06***	6.47	2.88–14.55	0.2	2.72	0.58–12.76
SRPX	cg16626088	2.13E−08***	26.69	8.46–84.23	0.99	1.01	0.090–11.53
SRPX	cg14759284	4.31E−13***	6	3.70–9.74	0.98	0.99	0.30–3.24
SRPX	cg19736094	6.00E−13***	5.11	3.44–7.58	0.57	0.62	0.12–3.14
SRPX	cg17407511	< 2.00E−16***	4.82	3.36–6.91	0.38	2.11	0.39–11.28
SRPX	cg27485646	1.03E−11***	6.98	3.99–12.23	0.56	0.66	0.16–2.66
SRPX	cg03509565	8.40E−13**	8.04	4.54–14.23	0.073	5.25	0.85–32.27

Odds ratios and *p* values presented for methylation *M* values (logistic-transformed beta values)

Statistical significance indicated by asterisks: *0.01 < *p* < 0.05; ***p < 0.001

Next, we examined methylation of these proximal probes in all tumors from the TCGA cohort (*n* = 428) as possible discriminators for the aforementioned clinicopathological variables (Table 4, Additional file 16: Figure S10). All seven genes with proximal probes possessed at least one probe able to significantly distinguish tumors of any GS (6, 7a, 7b, or 8+) from normal samples (Bonferroni-adjusted Dunn test, *p* < 0.05). Furthermore, all genes except *MEIS2* and *ASB2* possessed at least one methylation probe able to significantly distinguish tumors based on recurrence (Mann-Whitney *U* test, *p* < 0.05). Kaplan-Meier analysis of these significant probes revealed that high methylation levels at three *NRG1* probes were significantly associated with poor outcome in terms of recurrence-free survival (Fig. 5b). Two more probes in *NRG1* (cg00614182, log-rank *p* value = 0.0544) and *ETNK2* (cg20136584, log-rank *p* value = 0.0693) were trending for significance.

### *TET2*-target gene *ASB2* exhibits localized and specific gain of promoter methylation in prostate tumors within the *TET2*-target site

As *ASB2* was unique among the seven candidate genes in exhibiting a differential methylation pattern in its proximal methylation probes in the TCGA as compared to our KOs, we selected this gene for further validation to assess the specificity and accuracy of the *TET2*-directed methylation changes observed in our cell line model.

Following validation of *ASB2* methylation and expression data from sequencing via MBD-qPCR and RT-qPCR, respectively, in both KO and parental cells (Additional file 17: Figure S11 and Additional file 18: Figure S12), we examined methylation levels of our TET2-KO-specific DMR in an independent, limited series of primary patient samples comprised of matched normal and tumor tissues (*n* = 19 per group).

**Fig. 7:**
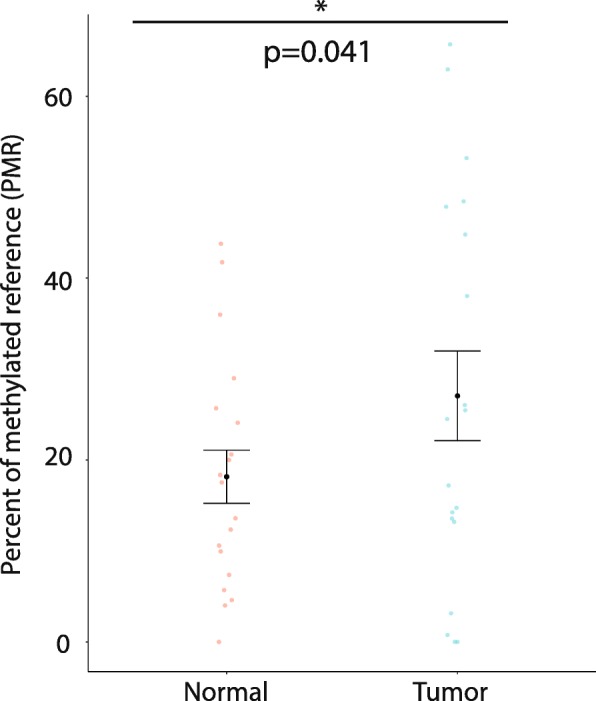
Promoter *ASB2* region gaining methylation in knockouts exhibits hypermethylation in prostate tumor samples compared to matched normal prostate. Scatterplot depicting increased methylation in matched tumor and normal samples (*n* = 18 per group), with mean and SEM depicted with error bars. Significance determined by paired Wilcoxon signed-rank test with continuity correction

We found that, in concordance with the results observed in our *TET2* KO models and in contrast to our observations in the TCGA dataset, methylation of the *ASB2* promoter region was significantly increased in tumor samples as compared to normal (*p* = 0.04067, paired Wilcoxon signed-rank test, Fig. 7). This observation underscores both qualitative and quantitative differences occurring in methylation marks within the *TET2*-targeted *ASB2* gene region.

## Discussion

Multiple lines of evidence have implicated a role for dysregulation of the master epigenetic regulator *TET2* in PCa development and progression. Although *TET2* is less frequently mutated in primary PCa as compared to metastatic PCa, several factors have been hypothesized as potentially contributing to its loss of expression, including hypoxia, which deprives *TET* enzymes of the oxygen required for their dioxygenase activity, alterations in expression of *TET*-governing transcription factors such as high-mobility group AT-hook 2 (*HGMA2*), and repression of *TET* by oncogenic miRNAs [25, 28]. Furthermore, *TET2* exhibits high mutation rates (10–20%) and extensive loss of heterozygosity (~ 60%) in metastatic prostate tumors [21–23, 28]. Genome-wide association studies have also shown increased PCa risk linked to an intergenic *TET2*-proximal SNP (rs7679673) [23, 29]. However, perhaps due to its low somatic mutation rates in primary prostate cancer, *TET2-*mediated changes have not been systematically investigated as potential drivers of cancer development. Therefore, we performed *TET2* KO in normal prostate RWPE-1 cells to identify targets of *TET2* mediation that may be important in prostate carcinogenesis.

Of the two different KOs, CR1 (the heterozygous knockout) showed unique methylation targeting of 487 genes, while the homozygous knockout model CR2 had 2682 uniquely methylated genes. *TET2* haploinsufficiency is enough to alter cellular properties and contribute to hematological malignancies, possibly due to a decrease in its catalytic activity [26, 30]. However, as complete loss of *TET2* protein is observed in both KOs, these findings suggest that certain TET2 mutations may cause a dominant negative phenotype. The usage of alternative compensatory mechanisms (such as increased activity of other *TET* enzymes) may explain the differential profiles between the two KOs; however, further studies are required to determine whether the type of *TET2* mutation influences the use of alternative 5hmC pathways.

Interestingly, we found that biological pathways affected by methylation gain in *TET2* KOs overlapped with intergenic 5hmC gain that we previously reported in PCa cell lines [16]. Paradoxically, despite the extensive global loss of 5hmC in PCa, increases in intergenic 5hmC levels were found to occur in genes related to inflammation and cellular adhesion—the same pathways enriched among silenced genes from our knockout model—resulting in downregulation of immune and adhesive functions. These observations suggest that not only are these critical functions governed by *TET2* dysregulation, but also that intergenic 5hmC and promoter 5mC gain may work synergistically to downregulate the same genes and pathways in cancer.

Intriguingly, out of seven key *TET2*-governed genes identified from our model exhibiting differential methylation in normal versus tumor samples in the TCGA at *TET2*-KO DMS-proximal probes, only one—*ASB2*—showed significantly *decreased*, rather than increased, methylation levels in cancer. However, the other target genes possessed probes either within the peaks identified through our methylation sequencing strategy (*ETNK2*, *MEIS2*, *NRG1*, *NUDT10*) or closer to these DMSs than those for *ASB2* (at distances of 328 bp, 160 bp, or 22 bp for *ASB2*, *NTN1*, or *SRPX*, respectively), and examination of methylation in matched tumor and normal samples from an independent series indicated concordant and increased 5mC within the *ASB2 TET2*-KO DMS. Furthermore, *TET2*-KO differentially methylated sites in all candidate genes were highly conserved in primates, ranging from 71.4% (5/7) of CpGs exhibiting conservation among at least three primate species in *ASB2* to 100% conservation for all CpGs in *ETNK2*, *MEIS2*, *NRG1*, and *SRPX*. This sequence conservation suggests that these *TET2*-mediated regions may possess a functionally important role.

Methylation of *ETNK2*, *NTN1*, and *NUDT10* was increased in ERG-fusion negative tumors, which is concordant with the significant loss of *TET2* expression in these tumor samples. The increased methylation of *MEIS2* and *NRG1* in ERG-positive tumors indicates that TMPRSS2-ERG fusion impacts the methylation status of these genes. However, expression of all seven candidate genes was also found to be independent of ERG fusion status in prostate tumors, which may indicate that their expression is influenced more strongly by *TET2* status than by mutation subtype in PCa. Taken together, these findings suggest that *TET2* loss results in highly localized region-specific, rather than widespread, changes in methylation at conserved regions within target gene promoters, which may have profound effects on gene expression.

Several prior studies have indicated the functional importance of our candidate gene panel in cancer development and progression. All candidate genes except ethanolamine kinase 2 (*ETNK2*) have been specifically linked to PCa in some manner. The cell migration-critical gene netrin 1 (*NTN1*), cellular adhesion molecule neuregulin 1 (*NRG1*), and the putative tumor suppressor sushi repeat-containing protein X-linked (*SRPX*) exhibit downregulation in prostate cancer as compared to normal prostate or benign prostate hyperplasia tissue [30–33], and evidence for association between the expression of ubiquitin ligase *ASB2* and PCa progression is established in the literature [34, 35]. Intriguingly, although we found *NRG1* probe methylation status to be significantly associated with recurrence-free survival by Kaplan-Meier analyses, its expression was not, even though both *NRG1* expression and methylation were associated with *TET2* expression status. This may indicate that candidate gene expression may be influenced in a methylation-independent manner (such as *TET2*-mediated O-GlcNAcylation); however, further studies are required to elucidate the mechanisms of this relationship.

Loss of the transcriptional regulator *MEIS2* is not only associated with recurrence and worse survival in PCa patients [36], but is also associated with the development of castration-resistant prostate cancer due to its inhibition resulting in constitutive activation of nuclear factor kappa-light-chain-enhancer of activated B cells (NF-κB) signaling [37]. Although expression of nudix hydrolase 10 (*NUDT10*) has not yet been examined in the context of PCa, the rs5945572 risk SNP within this gene is significantly associated with increased risk of PCa in Caucasian, African, and Asian ethnic groups, functionally implicating this gene in prostate cancer [38]. Intriguingly, several of these genes are upregulated in other types of cancers, including gastric, ovarian, and breast cancers, indicating that the mechanisms by which they contribute to PCa may be unique. Overall, further exploration of the *TET2* KO DMRs within the target genes identified by this study may uncover new insight into the mechanisms of carcinogenic changes in PCa.

In conclusion, we have demonstrated the utility of our *TET2*-knockout based candidate selection strategy in identifying significantly altered genes and potential markers of prostate carcinogenesis. Correlation of *TET2*-target genes with clinicopathological characteristics of PCa revealed a panel of seven promising candidates—*ASB2*, *ETNK2*, *MEIS2*, *NRG1*, *NTN1*, *NUDT10*, and *SRPX*—exhibiting discriminatory ability between tumor and normal samples and/or measures of PCa progression based on their 5mC and expression status. Lowered expression of three of these genes (*ASB2*, *NUDT10*, and *SRPX*), as well as increased methylation of *NRG1*, was significantly correlated with lower recurrence-free survival in PCa patients, showing the utility of these *TET2* targets as potential markers of disease. Validation of one such target gene, *ASB2*, indicated high specificity of *TET2-*mediated methylation, which may provide insight into the functionality of *TET2*-directed changes in PCa. Ultimately, future biological studies of epigenetic and transcriptomic disruption of candidate gene in cancer may allow us to not only identify new diagnostic and prognostic markers for PCa, but may also provide novel insights into the dynamic changes underlying development and progression in many different cancers exhibiting *TET2* dysregulation.

### Supplementary information

Supplementary information is available at *Clinical Epigenetics’* website.

## Materials and methods

### Cell culture and CRISPR knockout

Normal human prostate epithelial cell line, RWPE-1, was obtained from the American Type Culture Collection (ATCC, Manassas, Virginia). For CRISPR knockout, cells were transfected using Lipofectamine as per the manufacturer’s protocol with commercially available Sigma (St. Louis, Missouri) CRISPR plasmid expressing gRNA targeting the first coding exon of TET2, as well as Cas9 fused to GFP via a 2A linker peptide (gRNA target ID: HS0000114238; sequence: TTAGTAGCCTGACTGTTAA with TGG protospacer-associated motif). Forty-eight hours post-transfection, GFP-FACS was used to perform single-cell sorting of successfully transfected cells onto 96-well plates. Post-expansion, populations were assayed via Sanger sequencing (The Centre for Applied Genomics, Toronto) for the presence of indels. Off-target analysis was performed as described by Mali et al. [27]. RWPE-1 and knockout cells were cultured with keratinocyte serum-free medium (K-SFM) (Invitrogen, catalog #17005042), supplemented with 0.05 mg/mL bovine pituitary extract (BPE) and 5 ng/mL human recombinant epidermal growth factor (EGF). All cells were cultured as a monolayer and maintained in a humidified incubator at 37 °C with 5% CO_2_.

### Patient cohort

Radical prostatectomy tissue samples used in this study were comprised of treatment-naïve patients with PCa with matched normal tissue samples and were accrued for gene methylation analysis as per REB guidelines. Gleason score 7 samples were collected from October 2002 to July 2007, with a median follow-up time of 5.04 years (ranging from 3.88 to 9.79 years). Full clinical information for these patients can be found in Additional file 19: Table S7.

### DNA and RNA extraction

DNA was extracted from cell pellets using the QIAamp DNA Mini Kit (Qiagen, catalog #51306) following the manufacturer’s protocol. Whole RNA was extracted via TRIzol method as per the manufacturer’s protocol (Thermo Fisher, catalog #15596026) and was cleaned up using the RNeasy Mini Kit (Qiagen, catalog #74104).

### Whole-methylome and whole-transcriptome sequencing: MBD-Seq and RNA-Seq

2.5 μg sheared genomic DNA from RWPE-1 and CR1/CR2 knockout cells was enriched for 5mC with the MethylMiner kit (Invitrogen, catalog #ME10025) as previously described [16]. Enriched DNA was submitted in duplicate along with unenriched input controls for library preparation (NEBNext® ChIP-seq Library Prep Reagent Set for Illumina) and high-throughput sequencing using the HiSeq 2500 (Illumina, San Diego, California) at The Centre for Applied Genomics. Each library generated approximately 75 million paired-end reads. RNA library preparation for each of the above cell lines was performed following the NEB NEBNext Ultra Directional Library Preparation protocol (poly-A mRNA) on 200 ng total RNA. Libraries were sequenced in duplicate using the HiSeq 2500 at The Centre for Applied Genomics, with each library generating approximately 30 million paired-end reads.

### Bioinformatics analysis of MBD-Seq

Trimmed reads were mapped to hg19 using Bowtie2 (v2.2.1). [39] Repitools (v 1.12.1) was used to evaluate bound, enriched sample enrichment compared to input (Additional file 20: Figure S13). Significantly enriched regions of methylation were identified using Model-based Analysis of ChIP-Seq algorithm (MACS v 2.0.10). [40] DiffBind (v 1.12.2) was used to derive consensus peaks based on the presence of a peak in at least one sample analyzed. Annotation was performed using ChIPpeakAnno R package (v.2.16.4) [41] and a customized version of Annovar program [42] with RefSeq genes to determine specific genomic features closest to the peaks.

### Bioinformatics analysis of RNA-Seq

Trimmed reads were aligned to the hg19 human genome using Tophat v2.0.11, and raw read counts were extracted using htseq-count v0.6.1p2, with only uniquely mapping reads being counted. Raw gene counts were then loaded and sample-normalized using DESeq v1.18.0, and MDS analysis was performed to determine sample separation (Additional file 21: Figure S14). Two-condition differential expression was done with edgeR v3.8.6, filtering for only genes that were expressed. Quality of raw and trimmed reads was assessed with FastQC v0.11.2 and FastQ-Screen v0.4.3, and read distribution and strandedness was assessed with RSeQC v2.3.7. The above two analyses were performed by The Centre for Applied Genomics (Toronto).

### Western blotting

Cells were lysed with RIPA buffer supplemented with protease inhibitors and incubated on ice for 10 min, then centrifuged at 14,000 RPM for 10 min at 4 °C. Fifty-microgram samples with SDS loading dye were boiled and run on a 7.5% SDS-PAGE gel. Proteins were detected by immunoblotting (using 5% milk in TBS-T as blocking reagent) with antibodies against *TET2* (Abcam, ab94580) or *Ku80* (Cell Signaling Technologies, C48E7) overnight at 4 °C Unedited Western blot images from Figure 1B are shown in Additional 22: Figure S15.

### qRT-PCR

Primers were designed for specific targeting of *ASB2*–001 (isoform 2) and *ASB2–*002 (isoform 1) (GrCh37) so that the shared reverse primer spanned the junctions between exons 4 and 5 in isoform 1 and exons 3 and 4 in isoform 2. The forward primer for isoform 1 was located in its second exon (which is unique to isoform 1), while the forward primer for isoform 2 was located within a 67-bp region in its first exon unique to isoform 2 (Additional file 2: Table S1). Reverse transcription was performed using the qScript cDNA SuperMix kit (Quantabio, catalog #95048–100), and expression levels of transcripts were analyzed using SYBR Green qPCR.

### Pathway analysis of MBD-Seq datasets correlated with RNA-Seq expression data

Promoter region was defined as − 1500 bp to + 500 bp from the transcription start site of a given gene. Promoter regions gaining methylation and losing expression in knockouts compared to RWPE-1 were listed, and pathway enrichment analysis was performed using the Genomic Regions Enrichment of Annotations Tool (GREAT). [43] The whole human genome was used as background.

### MethyLight

Genomic DNA from matched normal and tumors was bisulfite converted and subjected to the MethyLight assay as previously described [44], using *ALU* as a methylation reference (Additional file 2: Table S1). Each sample was analyzed in duplicate on the Applied Biosystems® 7500 Real-Time PCR System. Methylation levels were determined using the delta-delta Ct method, with supermethylated (CH3) DNA from EMD Millipore (catalog #S7821) as control. Genes with Ct for *ALU* exceeding a value of 30 were excluded from the analysis.

### Statistical analyses

Association between candidate gene expression or methylation and tumor versus normal status or outcome was analyzed using Kruskal-Wallis and Mann-Whitney *U* tests as part of the base “stats” package of R (v3.4.0). Bonferroni correction was applied by dividing 0.05 by the number of samples analyzed and using the resultant value as the confidence threshold. Receiver operator curve (ROC) analysis was used to assess the discriminatory ability of candidate gene expression between tumor and normal samples. ROC plots, confidence intervals (via DeLong’s test), and AUC values were generated using the ROCR package in R (v1.0.7). Sensitivity and specificity were calculated based on the identification of the optimal cutoff point from ROC curve analysis maximizing both values. Univariate and multivariate logistic regression analyses were performed for individual gene expression values via generalized linear model analysis in R, using Wald *p* values to determine significance, and odds ratios were calculated via the logistic display function of the epiDisplay package (v3.5.0.1).

X-tile software (v3.6.1) was used to split the TCGA dataset into two parts, and X-tile plots were used to determine the optimal cutoff point for prediction of biochemical recurrence in the training set (cutoff points generated automatically by X-tile software) [45]. This cutoff was applied to the validation set as generated by X-tile software, and Kaplan-Meier survival curves and log-rank *p* values were used to assess individual gene performance. *p* < 0.05 was used as the confidence threshold for the above analyses unless otherwise specified in the manuscript.

## Additional files

Additional file 1: **Figure S1.** CRISPR-TET2 guide RNA targets the first coding exon of TET2. Arrows indicate the CRISPR target site on gene diagram of the functional TET2 isoform 1, which truncates the protein before any functional domains are produced. (TIF 134 kb)

Additional file 2: **Table S1.** Primer and probe sequences used for CRISPR verification and methylation-specific qPCR. (XLSX 17 kb)

Additional file 3: **Figure S2.** Off-target analysis of CRISPR-TET2 clones (CR1 and CR2). Sanger sequencing chromatograms for the top two gene regions partially matching the CRISPR-TET2 gRNA sequence shows no off-target effects of CRISPR on the parental gene sequence for either CR1 (top) or CR2 (bottom). (TIF 7908 kb)

Additional file 4: **Figure S3.** Overexposed Western blot for TET2 protein in parental RWPE-1 and TET2-KO cells. Overexposed Western blot shows no detectable bands for either TET2 isoform for CR1 or CR2 knockouts as compared to parental RWPE-1 cells. (TIF 4414 kb)

Additional file 5: **Figure S4.** Heatmap showing methylation of all genes exhibiting increased promoter methylation in TET2-KOs as compared to RWPE-1 cells. Methylation gradient bar indicates gene-normalized methylation levels, ranging from highest (yellow) to lowest (dark blue). Heatmap was generated via unsupervised clustering and clusters RWPE-1 cells separately from knockouts. (TIF 29540 kb)

Additional file 6: **Figure S5.** Heatmap showing expression of all differentially expressed and downregulated genes in both TET2-KOs as compared to RWPE-1 cells. Expression gradient bar indicates gene-normalized expression levels, ranging from highest (yellow) to lowest (dark blue). Heatmap was generated via unsupervised clustering and clusters RWPE-1 cells separately from knockouts. (TIF 29524 kb)

Additional file 7: **Table S2.** Genes exhibiting both significantly increased promoter methylation and significantly lowered expression in CRISPR TET2-KO cells. (XLSX 26 kb)

Additional file 8: **Figure S6.** Heatmap showing expression of 780 genes which exhibited significant expression alterations in knockout cells as well as low-*TET2* expressing tumors, for all tumor (*n* = 423) and normal (*n* = 35) samples in the TCGA. Expression gradient bar indicates gene-normalized expression levels, ranging from highest (yellow) to lowest (dark blue). Heatmap was generated via unsupervised clustering. (TIF 36633 kb)

Additional file 9: **Table S3.** Characteristics of the 61 genes exhibiting significantly increased promoter methylation and lowered expression in TET2-KOs as well as significantly lowered expression in TCGA prostate tumors. (XLSX 18 kb)

Additional file 10: **Table S4.** Expression of candidate genes significantly associated with ERG fusion status. (XLSX 18 kb)

Additional file 11: **Figure S7.** Pathway enrichment analysis of genes losing expression in CRISPR-TET2 knockout cells. Visual depiction of key pathways identified from GREAT analysis of all genes silenced in both CR1 and CR2, using the whole genome as a background. (TIF 917 kb)

Additional file 12: **Figure S8.** X-tile analysis and Kaplan-Meier plots for prediction of biochemical recurrence-free survival in the TCGA cohort. Left: X-tile plots depict *χ*^2^ values for all possible data divisions, with brightness indicating strength of association and green coloration indicating a direct relationship. Black circles on the bottom bars for each graph depict automatically generated cut points maximizing the *χ*^2^ value in an auto-generated training set. Middle: Histogram depicting the number of patients in the auto-generated validation set below (blue) or above (gray) the cutoff point. Right: Kaplan-Meier plot showing recurrence-free survival in low-expressing (blue; below cutoff) or high-expressing (gray; above cutoff) groups for each gene. Log-rank *p* values are indicated on each graph. (EPS 3514 kb)

Additional file 13: **Table S5.** Association of candidate gene probe methylation with ERG fusion status. (XLSX 19 kb)

Additional file 14: **Table S6.** Analysis of candidate gene probes in an independent methylation array dataset (GSE73549). (XLSX 20 kb)

Additional file 15: **Figure S9.** Methylation array probe locations on candidate genes with respect to the *TET2-*knockout methylated site and transcription start sites. Numbers indicate the genomic location of each probe (blue circle), transcription start site (green triangle), or *TET2*-knockout differentially methylated site (red rectangle). Probes chosen are within 500 base pairs of *TET2*-KO sites except for *ASB2*, which includes two additional probes within the promoter region. (TIF 37076 kb)

Additional file 16: **Figure S10.** Heatmap showing methylation of seven candidate genes for all tumor (*n* = 428) and normal (*n* = 50) samples in the TCGA. Methylation gradient bar indicates gene-normalized methylation beta values, ranging from highest (yellow) to lowest (dark blue). Heatmap was generated via unsupervised clustering. (TIF 33437 kb)

Additional file 17: **Figure S11.** Methylation of *ASB2* is increased in both knockouts (CR1 and CR2) within the *TET2*-knockout cell differentially methylated site. Expression analysis performed using MethyLight methylation-specific qPCR (*n* = 3). (TIF 472 kb)

Additional file 18: **Figure S12.** Expression of both *ASB2* isoforms is lowered in both knockouts as compared to parental RWPE-1 cells. Expression analysis performed using qRT-PCR (*n* = 3). (TIF 1636 kb)

Additional file 19: **Table S7.** Clinicopathological characteristics of prostate tumor tissues used for methylation analysis. (XLSX 15 kb)

Additional file 20: **Figure S13.** Diagnostic enrichment plot of MBD-Seq samples from TET2-KO cells as compared to input controls. Enrichment diagnostic graphs comparing the curve of input (non-enriched) sample (blue and yellow lines) to biological replicates of CR1 (green and orange lines) and CR2 (purple and red lines), respectively. (TIF 12551 kb)

Additional file 21: **Figure S14.** Multidimensional scaling (MDS) plot depicting Euclidean distances (similarities) between RNA-sequencing samples. MDS plot shows relative similarities between RWPE-1, TET2-KO cells, and the prostate adenocarcinoma 22Rv1 cell line based on RNA sequencing results. (TIF 13562 kb)

Additional file 22: Proteins were detected by immunoblotting with antibodies. (TIF 118 kb)
