# Supplementary figures and images for: Exploring targets of TET2-mediated methylation reprogramming as potential discriminators of prostate cancer progression
Source: Clin Epigenetics. 2019 Mar 27;11:54. doi: 10.1186/s13148-019-0651-z (PMC6438015; doi:10.1186/s13148-019-0651-z)

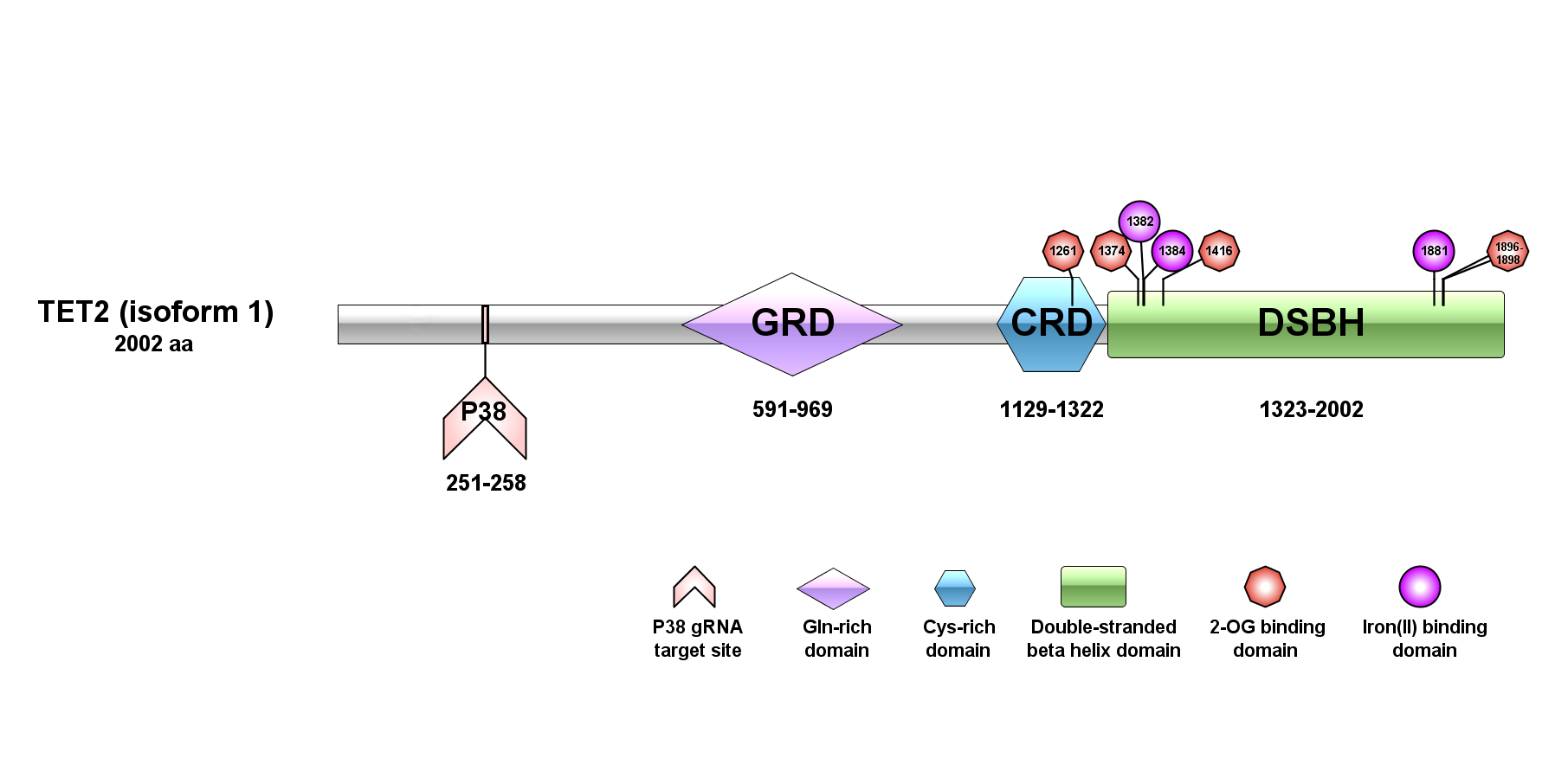

Supplement: Supplementary file 1 — Figure S1. CRISPR-TET2 guide RNA targets the first coding exon of TET2. Arrows indicate the CRISPR target site on gene diagram of the functional TET2 isoform 1, which truncates the protein before any functional domains are produced. (TIF 134 kb) [file 13148_2019_651_MOESM1_ESM.tif]

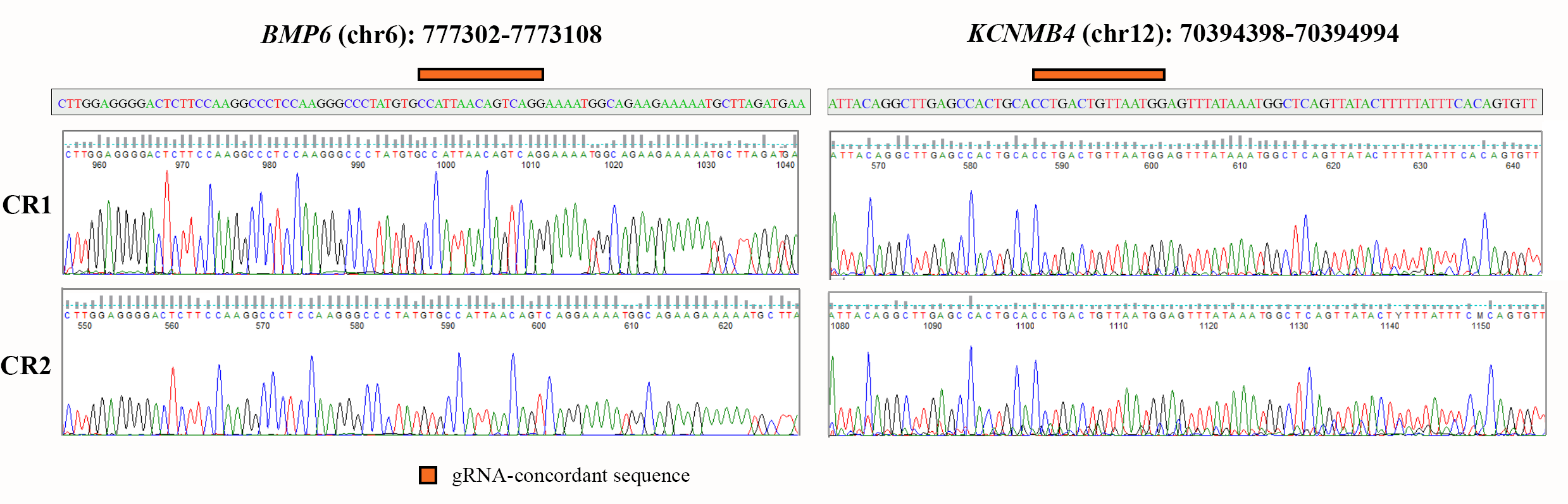

Supplement: Supplementary file 3 — Figure S2. Off-target analysis of CRISPR-TET2 clones (CR1 and CR2). Sanger sequencing chromatograms for the top two gene regions partially matching the CRISPR-TET2 gRNA sequence shows no off-target effects of CRISPR on the parental gene sequence for either CR1 (top) or CR2 (bottom). (TIF 7908 kb) [file 13148_2019_651_MOESM3_ESM.tif]

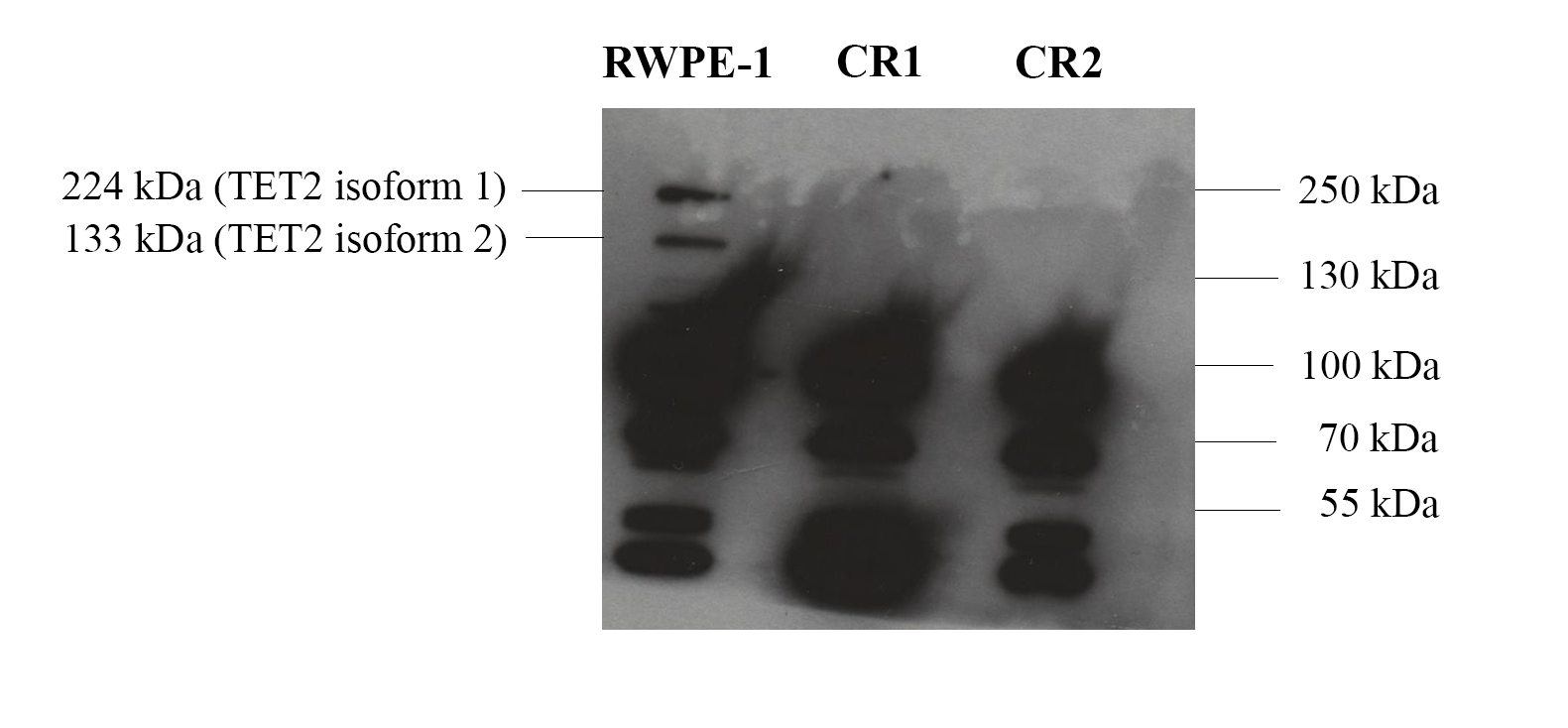

Supplement: Supplementary file 4 — Figure S3. Overexposed Western blot for TET2 protein in parental RWPE-1 and TET2-KO cells. Overexposed Western blot shows no detectable bands for either TET2 isoform for CR1 or CR2 knockouts as compared to parental RWPE-1 cells. (TIF 4414 kb) [file 13148_2019_651_MOESM4_ESM.tif]

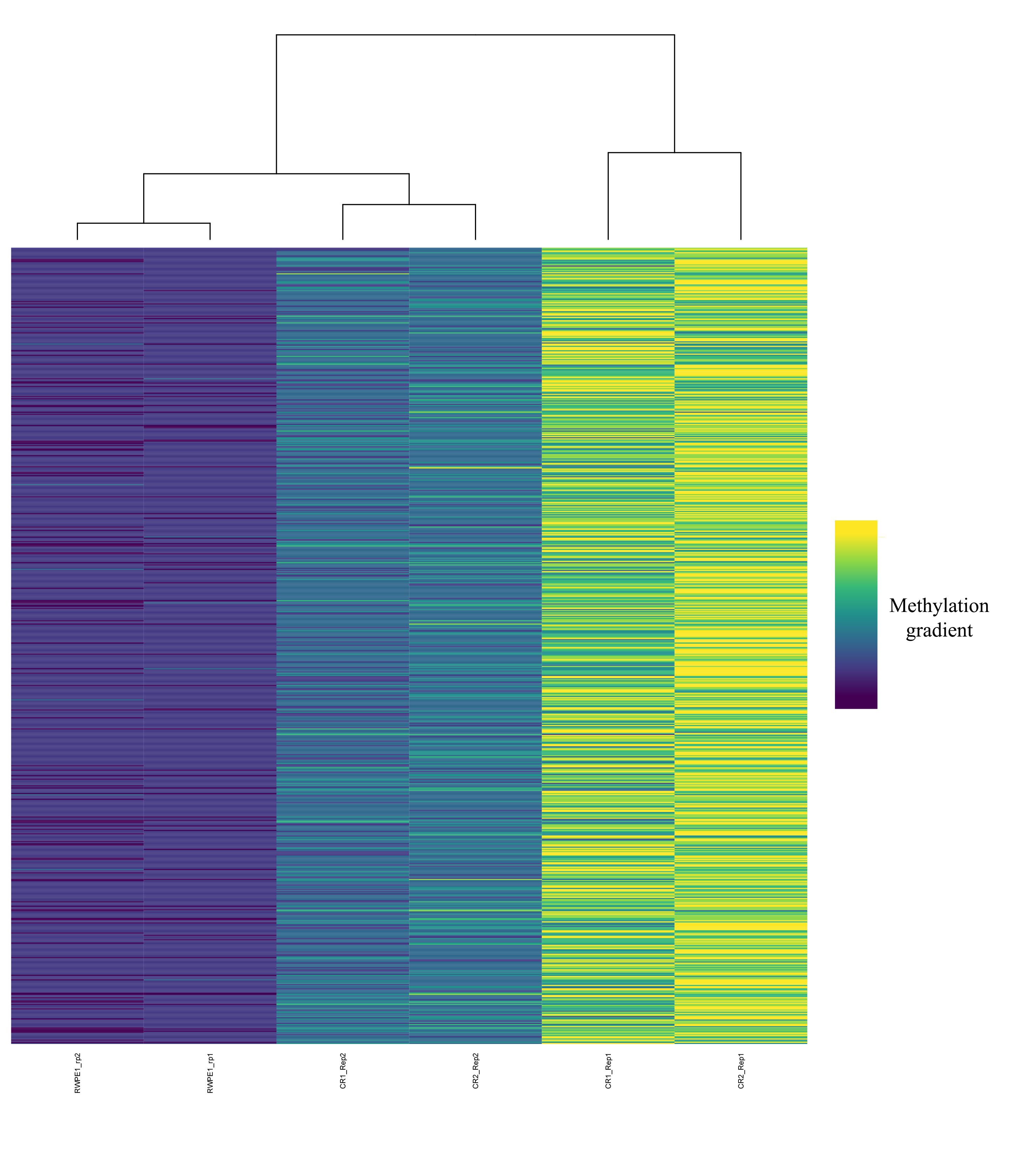

Supplement: Supplementary file 5 — Figure S4. Heatmap showing methylation of all genes exhibiting increased promoter methylation in TET2-KOs as compared to RWPE-1 cells. Methylation gradient bar indicates gene-normalized methylation levels, ranging from highest (yellow) to lowest (dark blue). Heatmap was generated via unsupervised clustering and clusters RWPE-1 cells separately from knockouts. (TIF 29540 kb) [file 13148_2019_651_MOESM5_ESM.tif]

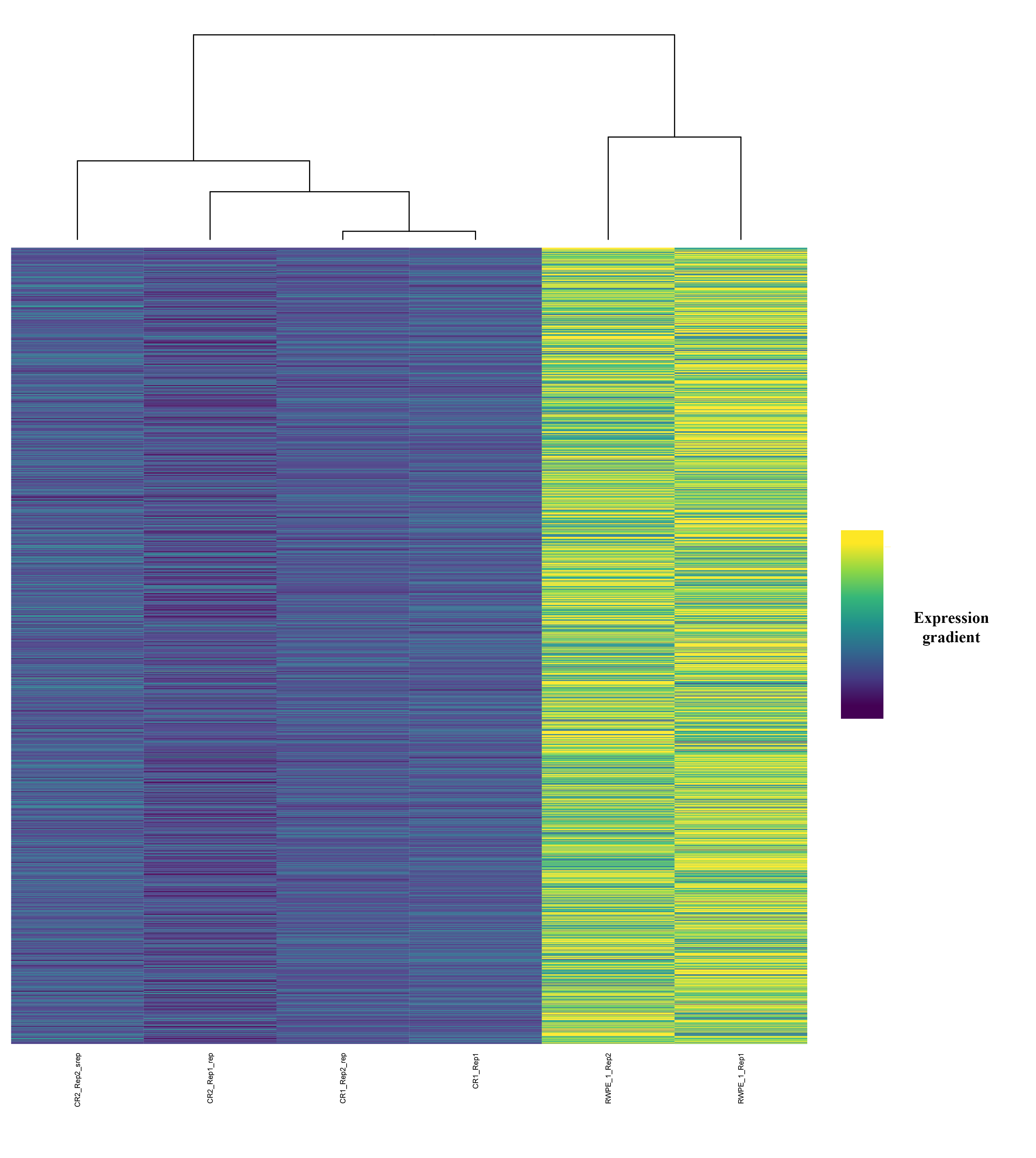

Supplement: Supplementary file 6 — Figure S5. Heatmap showing expression of all differentially expressed and downregulated genes in both TET2-KOs as compared to RWPE-1 cells. Expression gradient bar indicates gene-normalized expression levels, ranging from highest (yellow) to lowest (dark blue). Heatmap was generated via unsupervised clustering and clusters RWPE-1 cells separately from knockouts. (TIF 29524 kb) [file 13148_2019_651_MOESM6_ESM.tif]

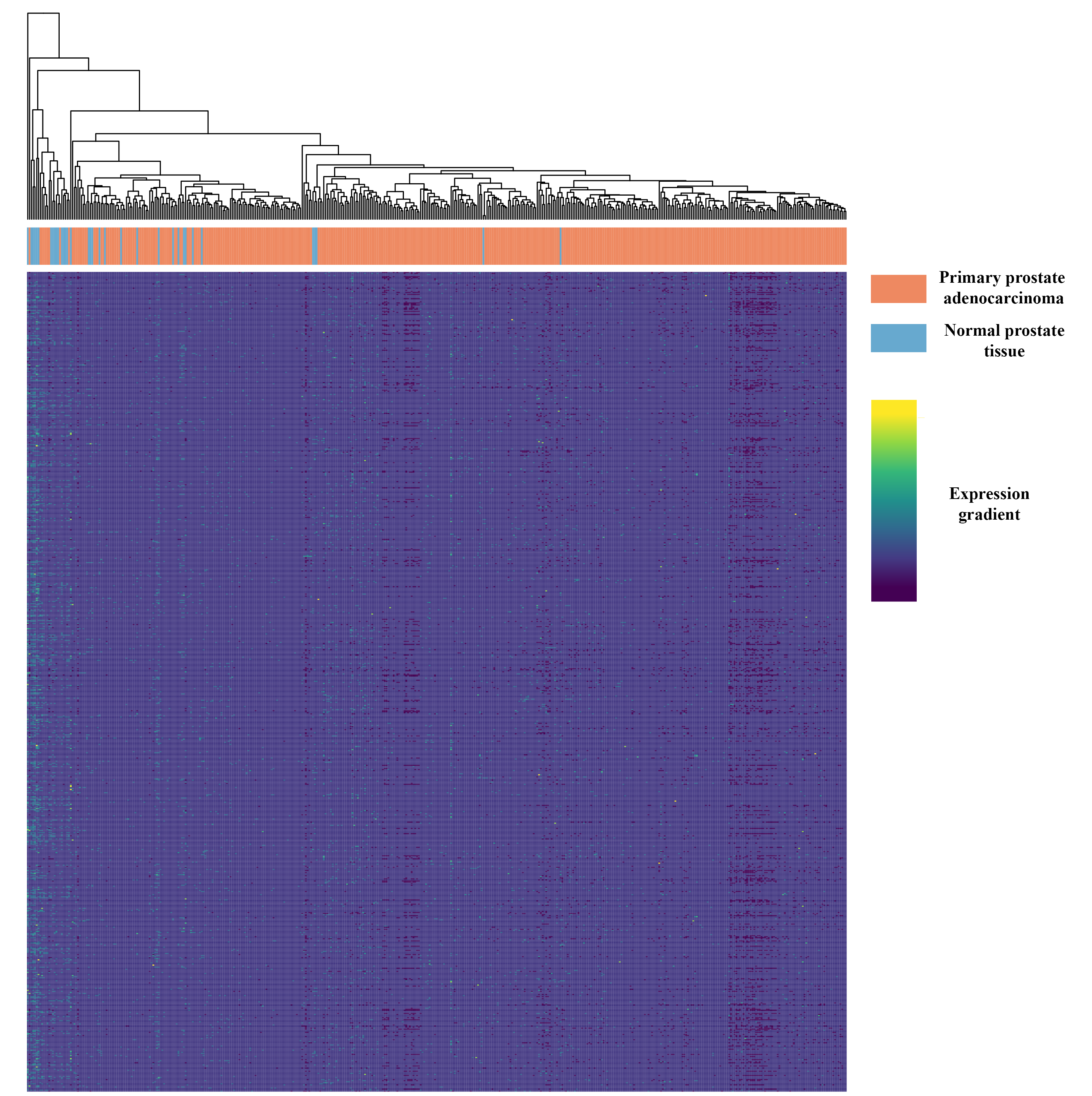

Supplement: Supplementary file 8 — Figure S6. Heatmap showing expression of 780 genes which exhibited significant expression alterations in knockout cells as well as low-TET2 expressing tumors, for all tumor (n = 423) and normal (n = 35) samples in the TCGA. Expression gradient bar indicates gene-normalized expression levels, ranging from highest (yellow) to lowest (dark blue). Heatmap was generated via unsupervised clustering. (TIF 36633 kb) [file 13148_2019_651_MOESM8_ESM.tif]

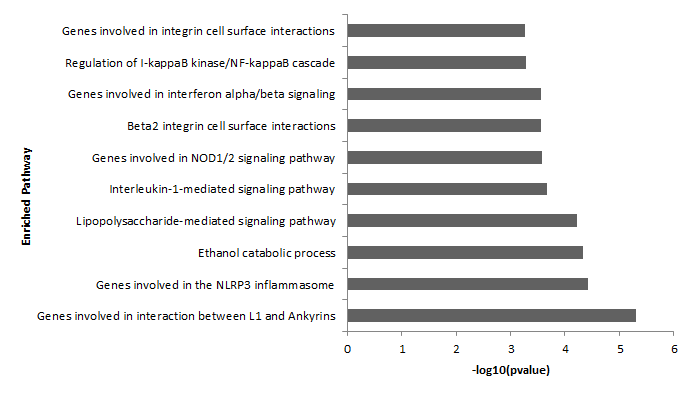

Supplement: Supplementary file 11 — Figure S7. Pathway enrichment analysis of genes losing expression in CRISPR-TET2 knockout cells. Visual depiction of key pathways identified from GREAT analysis of all genes silenced in both CR1 and CR2, using the whole genome as a background. (TIF 917 kb) [file 13148_2019_651_MOESM11_ESM.tif]

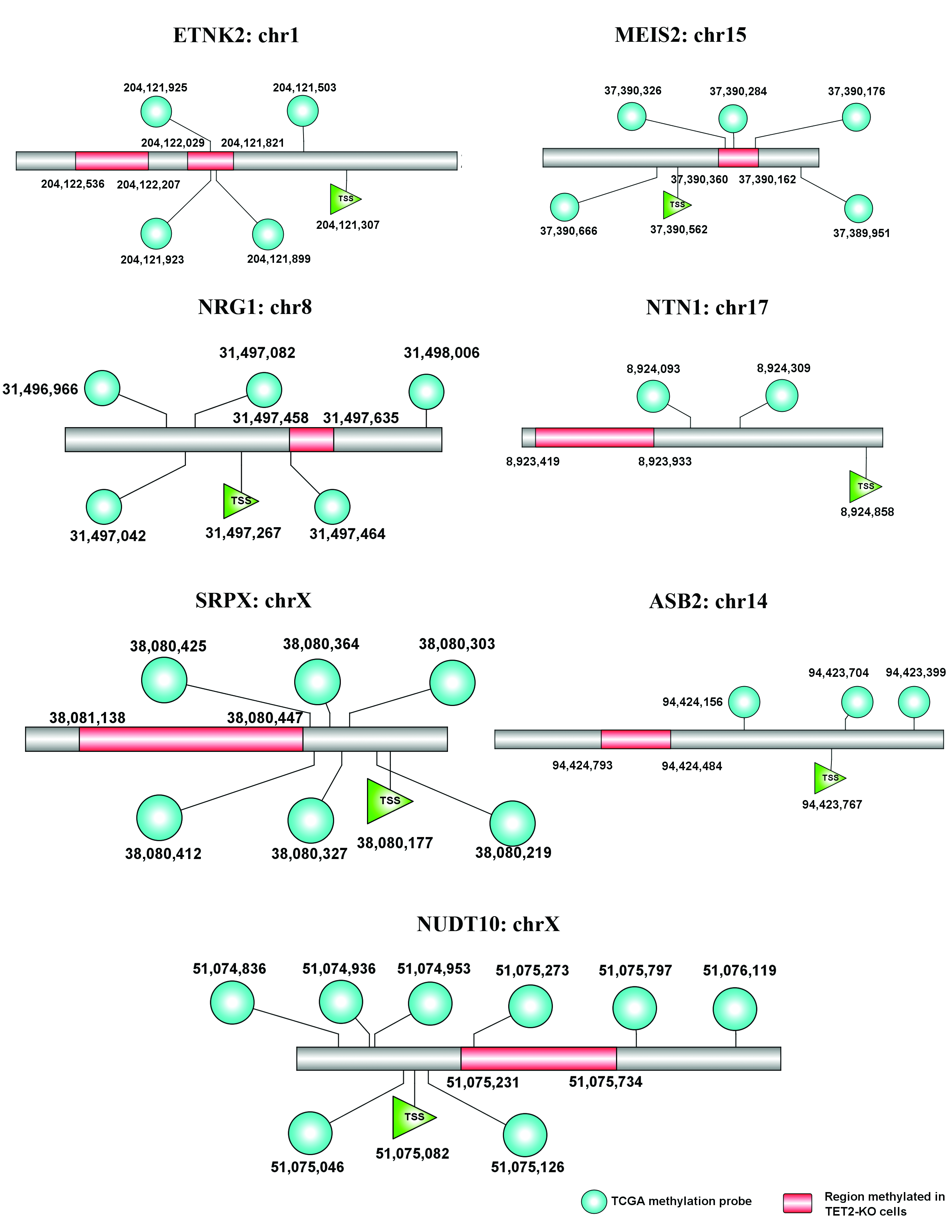

Supplement: Supplementary file 15 — Figure S9. Methylation array probe locations on candidate genes with respect to the TET2-knockout methylated site and transcription start sites. Numbers indicate the genomic location of each probe (blue circle), transcription start site (green triangle), or TET2-knockout differentially methylated site (red rectangle). Probes chosen are within 500 base pairs of TET2-KO sites except for ASB2, which includes two additional probes within the promoter region. (TIF 37076 kb) [file 13148_2019_651_MOESM15_ESM.tif]

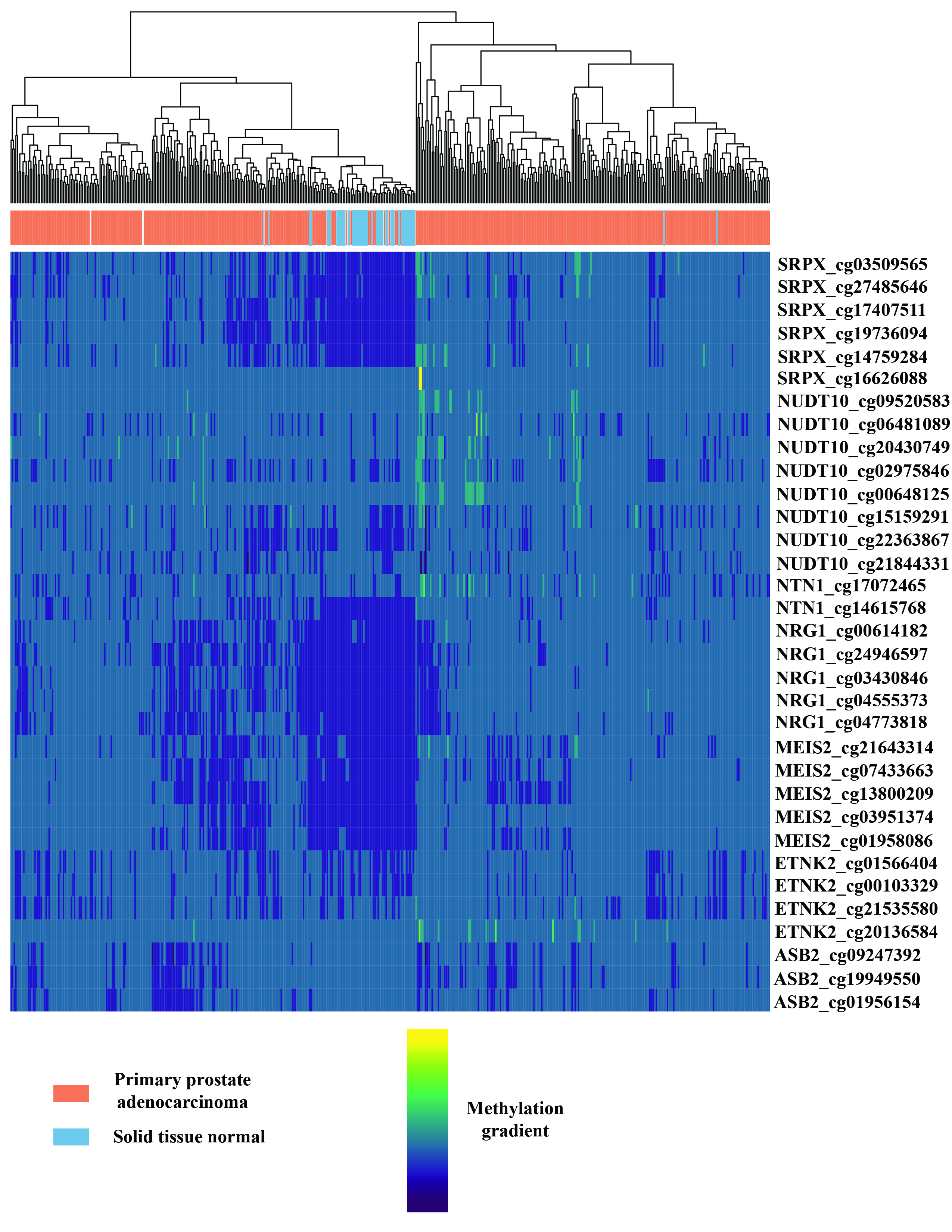

Supplement: Supplementary file 16 — Figure S10. Heatmap showing methylation of seven candidate genes for all tumor (n = 428) and normal (n = 50) samples in the TCGA. Methylation gradient bar indicates gene-normalized methylation beta values, ranging from highest (yellow) to lowest (dark blue). Heatmap was generated via unsupervised clustering. (TIF 33437 kb) [file 13148_2019_651_MOESM16_ESM.tif]

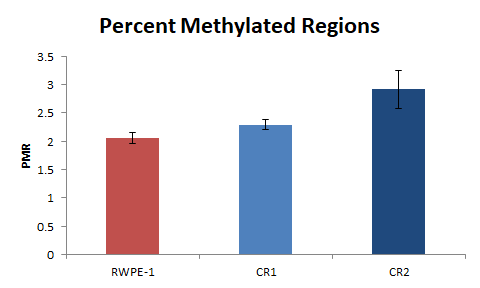

Supplement: Supplementary file 17 — Figure S11. Methylation of ASB2 is increased in both knockouts (CR1 and CR2) within the TET2-knockout cell differentially methylated site. Expression analysis performed using MethyLight methylation-specific qPCR (n = 3). (TIF 472 kb) [file 13148_2019_651_MOESM17_ESM.tif]

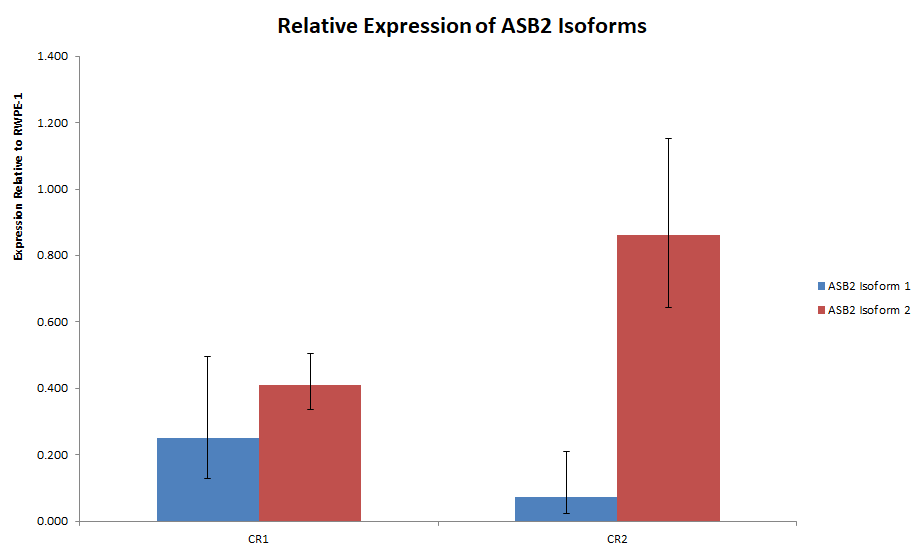

Supplement: Supplementary file 18 — Figure S12. Expression of both ASB2 isoforms is lowered in both knockouts as compared to parental RWPE-1 cells. Expression analysis performed using qRT-PCR (n = 3). (TIF 1636 kb) [file 13148_2019_651_MOESM18_ESM.tif]

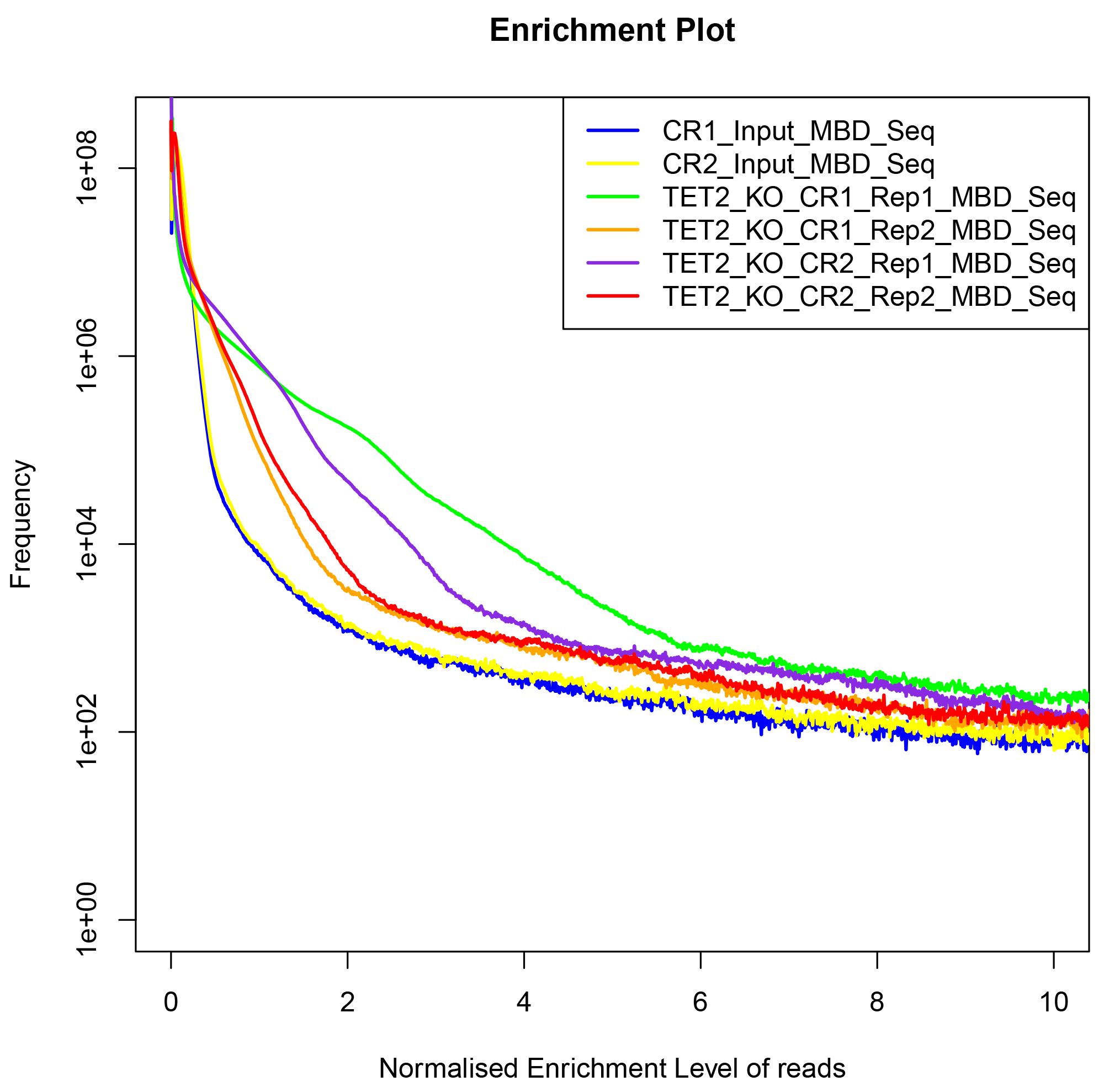

Supplement: Supplementary file 20 — Figure S13. Diagnostic enrichment plot of MBD-Seq samples from TET2-KO cells as compared to input controls. Enrichment diagnostic graphs comparing the curve of input (non-enriched) sample (blue and yellow lines) to biological replicates of CR1 (green and orange lines) and CR2 (purple and red lines), respectively. (TIF 12551 kb) [file 13148_2019_651_MOESM20_ESM.tif]

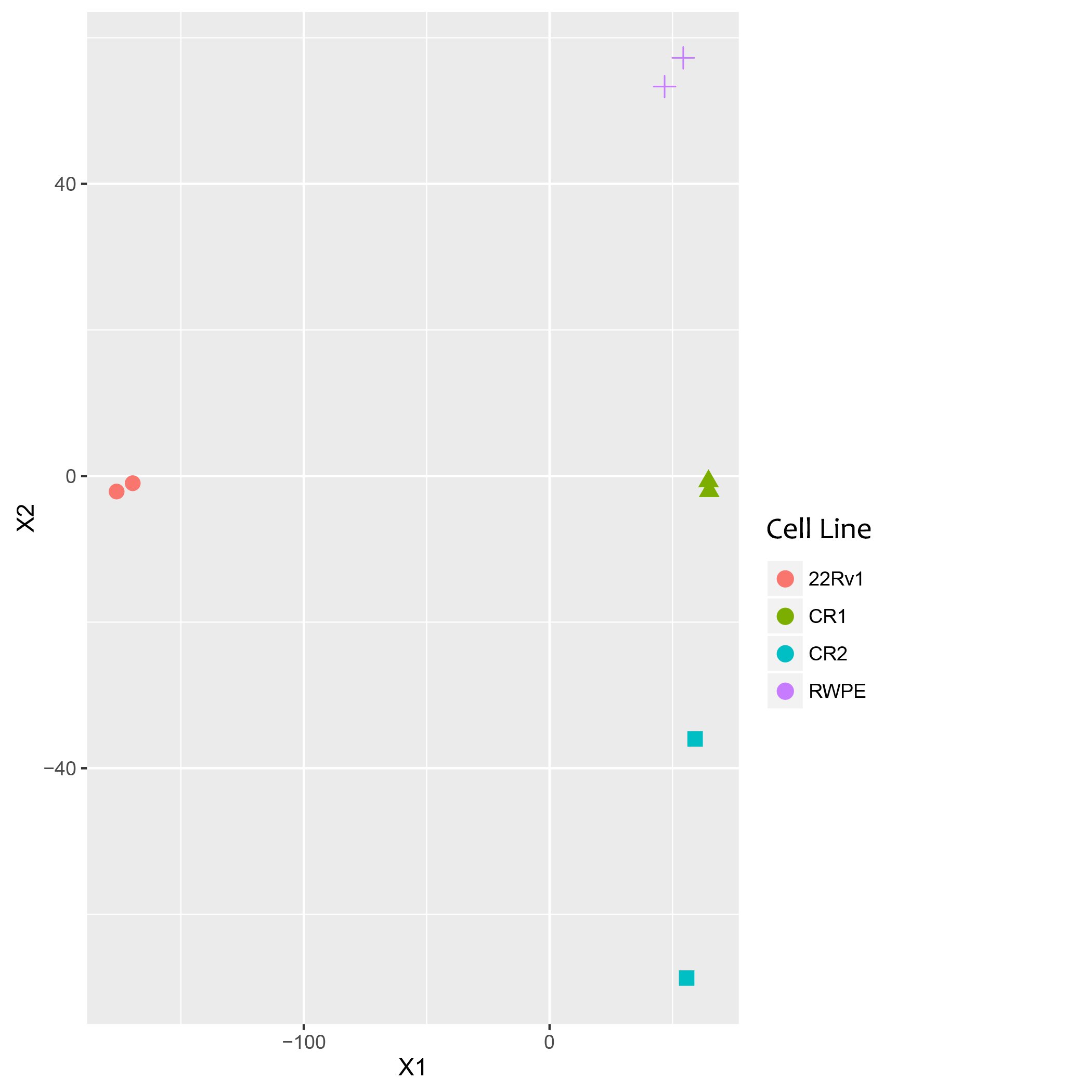

Supplement: Supplementary file 21 — Figure S14. Multidimensional scaling (MDS) plot depicting Euclidean distances (similarities) between RNA-sequencing samples. MDS plot shows relative similarities between RWPE-1, TET2-KO cells, and the prostate adenocarcinoma 22Rv1 cell line based on RNA sequencing results. (TIF 13562 kb) [file 13148_2019_651_MOESM21_ESM.tif]

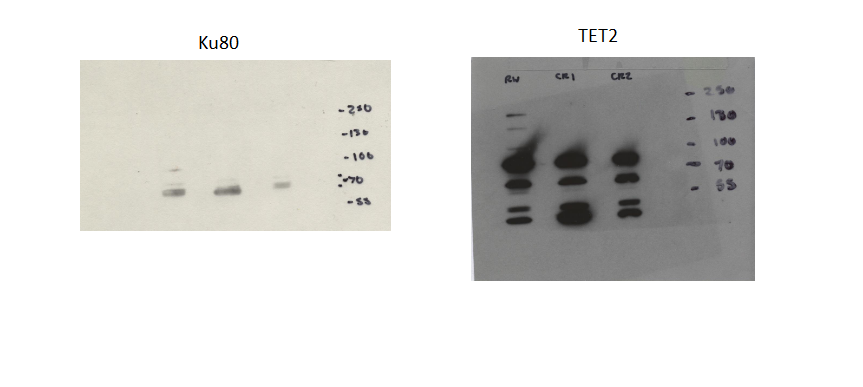

Supplement: Supplementary file 22 — Proteins were detected by immunoblotting with antibodies. (TIF 118 kb) [file 13148_2019_651_MOESM22_ESM.tif]
